# Epithelial pyroptosis-induced TREM1^+^ macrophages activate Th17 cells to accelerate oral mucosal inflammation

**DOI:** 10.1038/s41420-025-02853-7

**Published:** 2025-11-29

**Authors:** Qianhui Shang, Ziyuan Wang, Jiakuan Peng, Dan Yang, Weiqi Li, Xiaoyu Huang, Maofeng Qing, Hao Cheng, Jiaxin Liu, Hongxia Dan, Xin Zeng, Yu Zhou, Dunfang Zhang, Hao Xu, Qianming Chen

**Affiliations:** 1 State Key Laboratory of Oral Diseases, National Clinical Research Center for Oral Diseases, Research Unit of Oral Carcinogenesis and Management, Chinese Academy of Medical Sciences, West China Hospital of Stomatology, Sichuan University, Chengdu, Sichuan China; 2 Stomatology Hospital, School of Stomatology, Zhejiang University School of Medicine, Zhejiang Provincial Clinical Research Center for Oral Diseases, Zhejiang Key Laboratory of Oral Biomedical, Hangzhou, China; 3 School of Stomatology, North Sichuan Medical College, Nanchong, Sichuan China; 4 Department of Stomatology, The Second Affiliated Hospital of Chongqing Medical University, Chongqing, China; 5 Department of Biotherapy, State Key Laboratory of Biotherapy and Cancer Center, West China Hospital, Sichuan University, Chengdu, Sichuan China

**Keywords:** Mucosal immunology, Mucositis, Innate immunity

## Abstract

Chronic inflammation of the oral mucosa could affect daily living and even threaten systemic health. Unlike periodontitis, oral lichen planus, a common oral chronic inflammatory disease, has diverse clinical manifestations and can progress to malignancy. Hence, this study aimed to investigate the mechanism of oral chronic inflammation using single-cell RNA sequencing (scRNA-seq), spatial transcriptome, a large clinical follow-up cohort with bulk RNA sequencing, cytological experiments, and multiplex immunohistochemistry. We found that epithelial pyroptosis-induced triggering receptor expressed on myeloid cell-1 (TREM1)^+^ macrophages activated pathogenic T helper cell 17 via interleukin-1β, to spur the inflammatory development of oral mucosal epithelium. Besides, we established a spatiotemporal interactional online database, Oral-Gut Axis Mucosal Immune Atlas (ORGUAMIA), to uncover the extensive pro-inflammatory role of epithelial pyroptosis-induced TREM1^+^ macrophages in chronic digestive tract disorders. In summary, this study highlights the role of epithelial pyroptosis-induced TREM1^+^ macrophages accelerating mucosal epithelial inflammation and offers ORGUAMIA as a tool for researchers using scRNA-seq and spatial transcriptome without technological barriers.

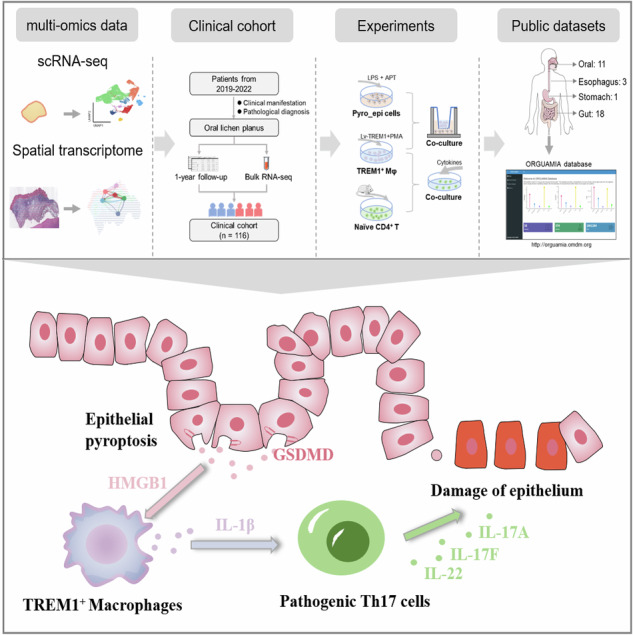

## Introduction

Oral mucosa is a pivotal barrier defense against harmful pathogens and ongoing mechanical damage. Chronic inflammation of the oral mucosa could affect normal daily life and even threaten systemic health, exemplified by conditions such as periodontitis and oral lichen planus (OLP), which are challenging to treat because of their high incidence, leading to a high disease burden [1–4]. Remarkably, oral mucosa is a crucial indicator and mirror of systemic health. Recent studies revealed that aberrant T helper cell 17 (Th17 cell) from oral mucosal chronic inflammation drives the pathogenesis of gut chronic inflammatory diseases through the oral-gut axis [5, 6]. Consequently, elucidating the immune mechanism behind chronic inflammation in the oral mucosa will enhance our understanding of the regulation of chronic inflammation in the digestive tract and systemic diseases.

With growing awareness of chronic oral mucosal inflammation, there has been significant progress in understanding the mechanisms of periodontitis. However, compared to periodontitis, there is still limited understanding of OLP, a common chronic inflammatory disease of the oral mucosa associated with systemic diseases that can progress to malignancy [7], with a global prevalence of 0.5–3% [8]. The clinical manifestations of OLP are diverse, with a malignant transformation rate of approximately 0.8–1.5% [8]. The etiology of OLP, commonly associated with T-cell mediation, remains unclear. Our previous study confirmed that CD8^+^ tissue-resident memory T cells accelerate oral mucosal epithelial inflammation via cytokine network [9]. Ongoing studies have increasingly acknowledged the role of innate immunity in chronic inflammation [5, 10, 11].

Macrophage (Mφ), as the first defense of innate immunity, is a bridge between innate immunity and adaptive immunity [12]. Mφ is recruited by pyroptosis of intestinal epithelial cells to accelerate intestinal inflammation [13]. Moreover, triggering receptor expressed on myeloid cells 1 (TREM1)^+^ Mφ are key pathogenic cell subsets that significantly amplify chronic inflammation in inflammatory bowel disease (IBD) [14, 15]. In addition, our previous study reported that macrophages, represented by innate immunity, are associated with inflammation of the oral mucosal epithelium [16]. Wu et al. [17] found TREM1, an amplifier of innate immune responses, could induce pro-inflammatory Mφ polarization to exacerbate periodontal inflammation; however, the intercellular regulatory mechanism of Mφ/innate immunity in chronic inflammation of the oral mucosa requires further clarification.

Hence, this study employs OLP as a research model of oral chronic inflammation to explore the immune mechanism of oral mucosal inflammation. We integrated single-cell RNA sequencing (scRNA-seq), spatial transcriptome, a large clinical follow-up cohort with bulk RNA-seq, cytological experiments, and multiplex immunohistochemistry (mIHC) to comprehensively elucidate that epithelial pyroptosis-induced TREM1^+^ Mφ activate pathogenic Th17 cells via interleukin-1β (IL-1β) to amplify oral mucosal inflammation. Additionally, we established a spatiotemporal interactional online database, Oral-Gut Axis Mucosal Immune Atlas (ORGUAMIA), to reveal the extensive pro-inflammatory role of epithelial pyroptosis-induced TREM1^+^ Mφ in chronic digestive tract disorders. Collectively, this study emphasizes how epithelial pyroptosis-induced TREM1^+^ Mφ accelerates the inflammation of the mucosal epithelium, with the ORGUAMIA database facilitating scRNA-seq and spatial transcriptome analysis without technological barriers.

## Results

### ScRNA-seq captured the tissue microenvironment of the oral mucosa with/without inflammation

**Fig. 1:**
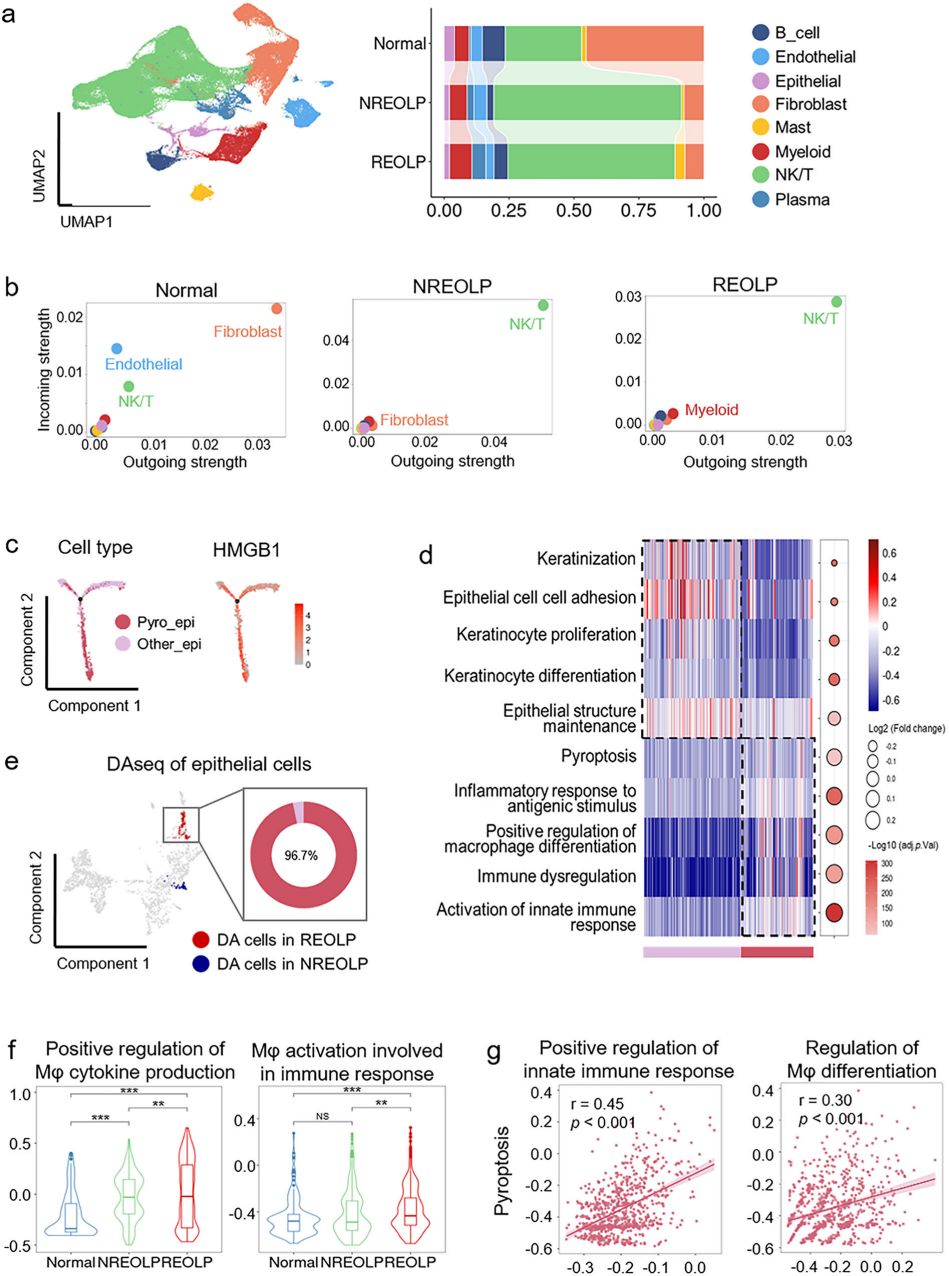
Single-cell RNA sequencing captured the tissue microenvironment of oral mucosa with/without inflammation and revealed pyroptosis of epithelial cells may promote the inflammatory development of oral mucosal epithelium. **a** Left, UMAP plot of 128,694 cell colors by cell types. Right, histogram showing the cell proportions of all cell types with disease subtypes. **b** Scatterplots exhibiting the intercellular interaction strength colored by cell types in normal (left), NREOLP (middle), and REOLP (right). **c** Developmental trajectory of epithelial cell subtypes (left) and HMGB1 (right). **d** Heatmap and dot plot revealing the biological function characteristics of epithelial cell subtypes. Dot size, the log_2_(fold change) of signaling pathways; color scale, −log_10_(adjust p-value) of signaling pathways. **e** UMAP plot and donut plot displayed the distribution and cell proportions of the differential abundance (DA) epithelial cells in the inflammatory development of oral mucosal epithelium. Red: DA epithelial cells in REOLP. Blue: DA epithelial cells in NREOLP. **f** Violin plot showing the change of signaling pathways in the inflammatory development of oral mucosal epithelium. Note: **p < 0.01; ***p < 0.001; NS: not statistically significant. **g** Scatterplots showin**g** the correlation of the “Pyroptosis” signaling pathway and the signaling pathways of “Positive regulation of innate immune response” (left) and “Regulation of Mφ differentiation” (right) in pyroptotic epithelial cells of REOLP.

ScRNA-seq was utilized to capture the tissue microenvironment in the normal oral mucosa (*n* = 3), non-recurrent erosive OLP (NREOLP, *n* = 4), and recurrent erosive OLP (REOLP, *n* = 6). After quality control, 128,694 cells were finally obtained from thirteen samples derived from human oral buccal mucosa. Eight major cell types were clustered and annotated (Figs. 1a and S1a).

In normal oral mucosa, fibroblasts comprised the highest cell proportion (45.3%) and played a major role in intercellular communication (Fig. 1a, b), consistent with a previous study [18]. In inflammatory oral mucosa, NK/T cells had the highest cell proportion (NREOLP: 72.2%, REOLP: 64.3%) and played a significant role in intercellular communication (Fig. 1a, b). Using DAseq [19], a differential abundant (DA) cell subpopulation detection algorithm in scRNA-seq data, NK/T cells were identified as significant DA cells in OLP (Fig. S1b). Myeloid cells were the major DA cells with the increased proportion and significantly enhanced intercellular communication during the inflammatory development of oral mucosal epithelium (Fig. 1a, b and Fig. S1c). This underscores the potential integral role of myeloid cells in the inflammatory development of oral mucosa and epithelial barrier damage.

### Epithelial pyroptosis may trigger aberrant innate immunity to accelerate oral mucosal inflammation

We first focused on epithelial cells, considering the damaged epithelial barrier. A cluster of epithelial cells exhibited high expression of pyroptosis-related markers (Caspase-1 [CASP1], CASP4, Gasdermin D [GSDMD], and high mobility group box-1 protein [HMGB1]; Fig. S1d). Therefore, we classified epithelial cells into pyroptotic epithelial cells and other epithelial cells (Fig. S1e).

Pyroptotic epithelial cells exhibited pro-inflammatory properties compared with other epithelial cells. They were located at the end of the epithelial cell differentiation trajectory and coincided with the cell differentiation trajectory of the inflammatory development in the oral mucosal epithelium (Fig. 1c and Fig. S1f). In addition, HMGB1, as a marker of pyroptosis, gradually increased at the end of the epithelial cell differentiation trajectory, while KRT14 had an opposite expression pattern (Fig. 1c and Fig. S1f). Meanwhile, the activated immune-related pathways in pyroptotic epithelial cells were upregulated (Fig. 1d).

Notably, pyroptotic epithelial cells were not only the major DA epithelial cells during the inflammatory development of oral mucosal epithelium (Fig. 1e) but the cell proportion was also elevated (Fig. S1g). In pyroptotic epithelial cells, the pathways of “positive regulation of Mφ cytokine production” (False discovery rate [FDR] < 0.001) and “Mφ activation involved in immune response” (FDR < 0.001) were significantly upregulated during the inflammatory development of oral mucosal epithelium (Fig. 1f). Additionally, the “Pyroptosis” pathway positively correlated with the “Positive regulation of innate immune response” (*p* < 0.001) and “Regulation of Mφ differentiation” pathways in REOLP (*p* < 0.001; Fig. 1g). This suggests that pyroptotic epithelial cells may activate the innate immune response, especially in Mφ, during the inflammatory development of oral mucosal epithelium.

### TREM1^+^ Mφ, activated by epithelial pyroptosis, could spur T cell accumulation during the inflammatory development of oral mucosal epithelium

**Fig. 2:**
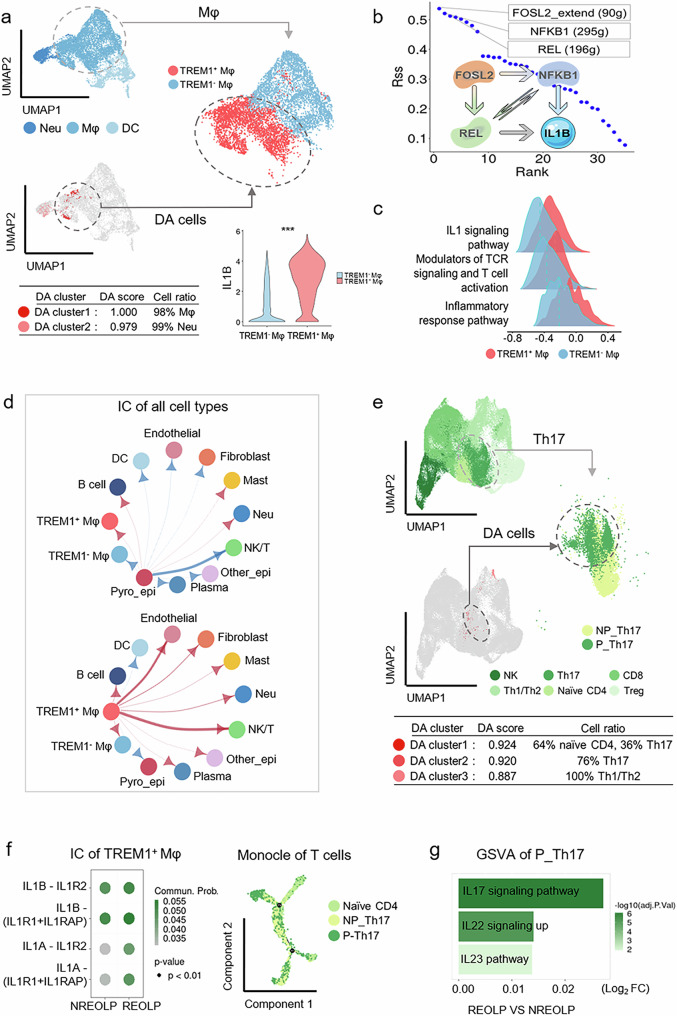
TREM1^+^ Mφ, may be triggered by pyroptotic epithelial cells, promoted pathogenic Th17 cell differentiation to exacerbate the inflammation of oral mucosal epithelium. **a** Top left, UMAP plot of 9542 cell colors by myeloid cell types. Gray circle: Mφ. Bottom left, UMAP plot displayed the differential abundance (DA) myeloid cells of the inflammatory development of oral mucosal epithelium. Red: DA myeloid cells in REOLP. Blue: DA myeloid cells in NREOLP. Gray circle: DA Mφ in REOLP. Top right, UMAP plot of 6,680 cell colors by Mφ types. Gray circle: DA Mφ in REOLP. Bottom right, violin plot of the expression of IL1B in different Mφ subtypes. **b** Scatterplot and network plot showing the active transcription factors of TREM1^+^ Mφ and the PPI of active transcription factors and IL-1β. **c** Ridge plots showed the differential expression of signaling pathways in TREM1^+^ Mφ and TREM1^-^ Mφ. **d** Circle plot of the intercellular communication (IC) from pyroptotic epithelial cells (top) and TREM1^+^ Mφ (bottom) to other cells in the inflammatory development of oral mucosal epithelium. Line width, the IC strength. Red arrow, the IC strength in REOLP was higher than in NREOLP. Blue arrow, the IC strength in NREOLP was higher than in REOLP. **e** Top left, UMAP plot of 75,336 cells colors by NK/T cell types. Gray circle: Th17 cells. Bottom left, UMAP plot displayed the DA NK/T cells of the inflammatory development of oral mucosal epithelium. Red: DA NK/T cells in REOLP. Blue: DA NK/T cells in NREOLP. Gray circle: DA Th17 cells in REOLP. Right, UMAP plot of 10,954 cell colors by Th17 cell types. Gray circle: DA Th17 cells in REOLP. **f** Left, dot plot of the IC from TREM1^+^ Mφ to naïve CD4^+^ T cells in IL1 signaling pathway in the inflammatory development of oral mucosal epithelium. Dot size, p-value; color scale, IC probability. Right, the developmental trajectory of T cell subtypes. **g** Bar plot exhibiting the upregulated signaling pathways of pathogenic Th17 cells in the inflammatory development of oral mucosal epithelium.

To confirm the above speculation, myeloid cells were sub-grouped into Mφ, neutrophils, and dendritic cells (Fig. 2a and Fig. S1h). Notably, the major DA cells in myeloid cells were Mφ specifically expressing TREM1 (Fig. 2a and Fig. S1i). Thus, Mφ was further classified as TREM1^+^ Mφ and TREM1^-^ Mφ (Fig. 2a).

TREM1^+^ Mφ was characterized by elevated IL1B expression (Fig. 2a) and had a significant cell proportion of M1 Mφ (82.1%; Fig. S1j). The active transcription factors of TREM1^+^ Mφ, including FOSL2, NFKB1, and REL, could directly or indirectly regulate downstream IL-1β based on the single-cell regulatory network inference and clustering (SCENIC) analysis and protein-protein interaction network (PPI) analysis (Fig. 2b). Moreover, the IL-1 signaling pathway was also upregulated in TREM1^+^ Mφ (Fig. 2c).

The proportion of TREM1^+^ Mφ increased during the inflammatory development of oral mucosal epithelium (Fig. S1k). Additionally, intercellular communication of pyroptotic epithelial cells was elevated to TREM1^+^ Mφ, neutrophils, mast cells, and B cells in the inflammatory development of oral mucosal epithelium (Fig. 2d). Fig. S1l shows the PPI between HMGB1, TREM1, and CD68, suggesting potential communication between pyroptotic epithelial cells and TREM1^+^ Mφ via HGMB1. Moreover, TREM1^+^ Mφ exhibited significant upregulation of the “Modulators of TCR signaling and T cell activation” signaling pathway and strongly communicated with NK/T cells during the inflammatory development of oral mucosal epithelium (Fig. 2c, d). These findings imply that TREM1^+^ Mφ, potentially activated by epithelial pyroptosis, may further affect NK/T during the inflammatory development of oral mucosal epithelium.

### Pathogenic Th17 cells exacerbated the oral inflammation induced by IL-1β secreted from TREM1^+^ Mφ

Next, NK/T cells were grouped as Th17 cells (including pathogenic and non-pathogenic Th17 cells), naïve CD4^+^ T cells, Th1/Th2 cells, Treg cells, CD8^+^ T cells, and NK cells. Of these, naïve CD4^+^ T cells and Th17 cells, especially in pathogenic Th17 cells, were the major DA NK/T cells and increased cell proportion during the inflammatory development of oral mucosal epithelium (Fig. 2e and Fig. S1m–o).

Intercellular communication analysis revealed an enhanced interaction from TREM1^+^ Mφ to naïve CD4^+^ T cells mediated by IL-1β during the inflammatory development of OLP. Furthermore, pseudotime analysis indicated that these naïve CD4^+^ T cells subsequently differentiated into pathogenic Th17 cells (Fig. 2f). Moreover, the IL-17, IL-22, and IL-23 signaling pathways were upregulated in pathogenic Th17 cells during the inflammatory development of oral mucosal epithelium (Fig. 2g), potentially recruiting immune cells to exacerbate the inflammatory response [20]. These findings suggest that pathogenic Th17 cells, possibly induced by IL-1β originating from TREM1^+^ Mφ, contribute to the inflammatory development of oral mucosal epithelium.

### The spatial atlas revealed the essential role of TREM1^+^ Mφ, epithelial pyroptosis, and pathogenic Th17 cells in oral mucosal inflammation

**Fig. 3:**
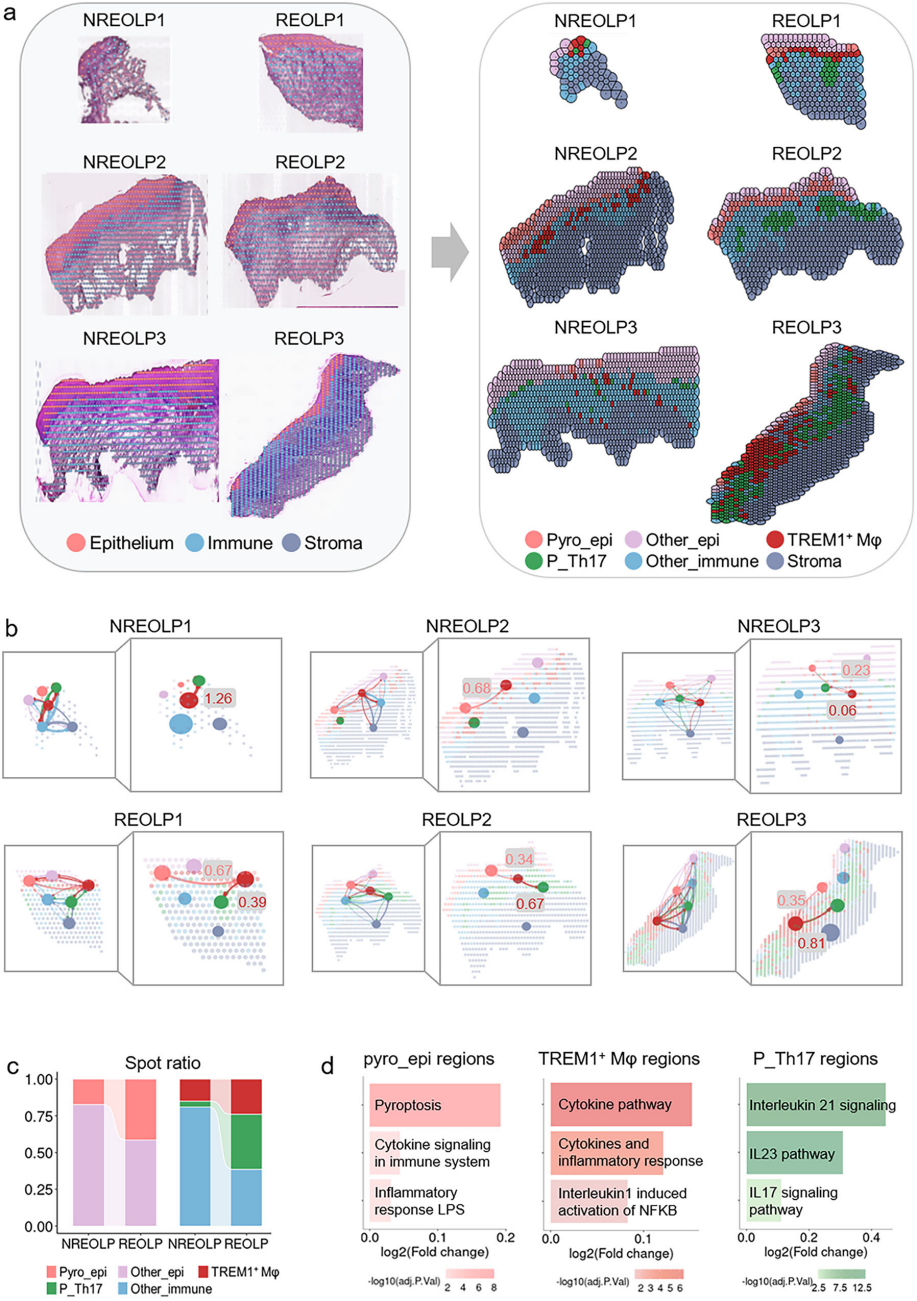
Spatial atlas of TREM1^+^ Mφ, pyroptotic epithelial cells, and pathogenic Th17 cells in the inflammatory development of oral mucosal epithelium. **a** Spatial scatter plot of tissue zones (left) and spatial distribution of cell types (right) in the inflammatory development of oral mucosal epithelium. **b** Spatial intercellular communication of different samples in the inflammatory development of oral mucosal epithelium. **c** Histogram showing the spot proportions of cell types in epithelial regions (left) and immune regions (right) during the inflammatory development of oral mucosal epithelium. **d** Barplots showing the upregulated signaling pathways of pyroptotic epithelial cell regions (left), TREM1^+^ Mφ regions (middle), and pathogenic Th17 cell regions (right) during the inflammatory development of oral mucosal epithelium.

To address the limitations of scRNA-seq, which lacks spatial locations, this study utilized spatial transcriptome analysis to explore the mechanism of the inflammatory development of oral mucosal epithelium from the spatiotemporal perspective. Six samples were separately divided into three tissue regions, including epithelial regions (CDH1, KRT6A, and KRT10), immune regions (PTPRC, ITGAM, and CD3D), and stromal regions (COL1A2, DCN, and VWF; Fig. 3a and Fig. S2a–f). The epithelial and immune regions of all samples were further integrated, sub-clustered, and annotated based on the above scRNA-seq data, resulting in the identification of pyroptotic epithelial regions, other epithelial cell regions, TREM1^+^ Mφ regions, pathogenic Th17 cell regions, and other immune cell regions (Fig. 3a). In addition, markers of pyroptosis, TREM1^+^ Mφ, and pathogenic Th17 cells were highly expressed in pyroptotic epithelial regions, TREM1^+^ Mφ regions, and pathogenic Th17 cell regions (Fig. S2g, h), respectively, confirming the accuracy of spot annotation.

The spatial atlas revealed that the spatial location of pyroptotic epithelial, TREM1^+^ Mφ, and pathogenic Th17 cell regions was closer during the inflammatory development of oral mucosal epithelium (Fig. 3a). Different cell regions exhibited complex spatial intercellular communication within inflamed oral mucosa. Moreover, the spatial intercellular communication from pyroptotic epithelial regions to TREM1^+^ Mφ regions and from TREM1^+^ Mφ regions to pathogenic Th17 cell regions was elevated during the inflammatory development of oral epithelium (Fig. 3b). Additionally, the proportion of spots and expression of markers collectively increased in pyroptotic epithelial regions, TREM1^+^ Mφ regions, and pathogenic Th17 cell regions during the inflammatory development of oral mucosal epithelium (Fig. 3c and Fig. S2i–l). The corresponding signaling pathways associated with these regions were upregulated (Fig. 3d), indicating their active pro-inflammatory function during the inflammatory development of oral mucosal epithelium. Interestingly, “Inflammatory response LPS” signaling pathway was upregulated in pyroptotic epithelial regions during the inflammatory development of oral mucosal epithelium (Fig. 3d). This spatial evidence confirms that epithelial pyroptosis-induced TREM1^+^ Mφ could activate pathogenic Th17 cells, thereby magnifying oral mucosal inflammation.

### The clinical cohort demonstrated that TREM1^+^ Mφ, epithelial pyroptosis, and pathogenic Th17 cells could increase the risk of the inflammatory development of oral mucosal epithelium

**Fig. 4:**
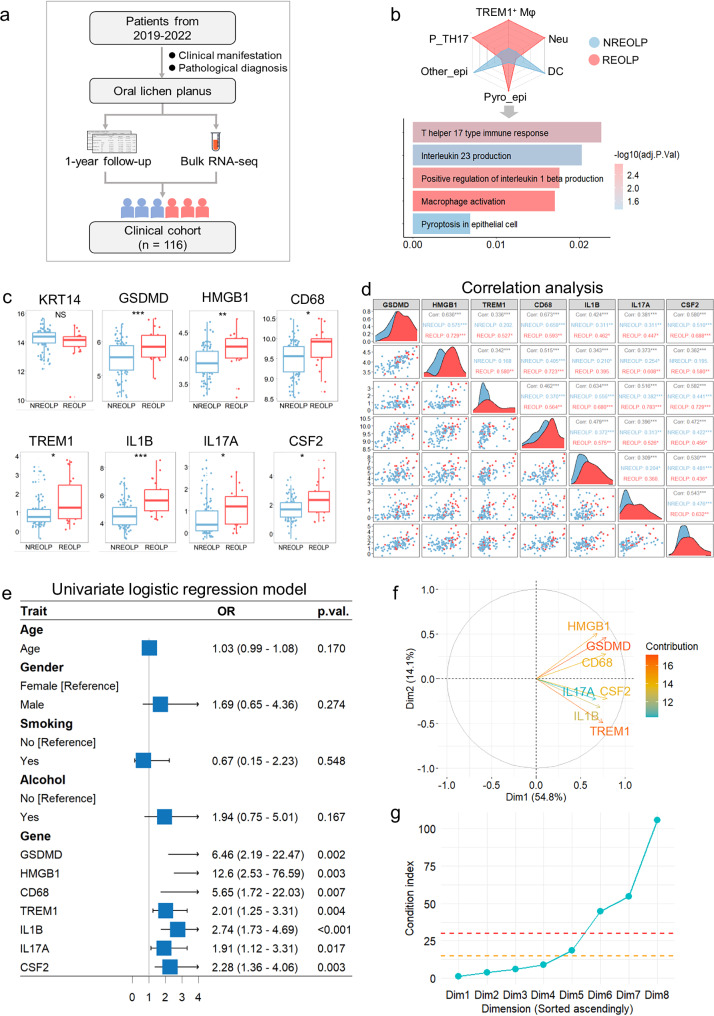
Clinical cohort demonstrated that TREM1^+^ Mφ, pyroptotic epithelial cells, and pathogenic Th17 cells could increase the risk of the inflammatory development of oral mucosal epithelium. **a** Flow chart of the establishment of the clinical follow-up cohort. **b** Top, radar plot of cell type fraction in the inflammatory development of oral mucosal epithelium based on BayesPrism deconvolution. Bottom, barplot of the upregulated signaling pathways in the REOLP through ssGSEA. **c** Box plots show the expression of cell markers in the inflammatory development of oral mucosal epithelium. **d** Density plots exhibiting the correlation of cell markers in pyroptotic epithelial cells, TREM1^+^ Mφ, and pathogenic Th17 cells in the inflammatory development of oral mucosal epithelium. Red: REOLP. Blue: NREOLP. **e** Univariate logistic regression model revealing the risk factors of the inflammatory development of oral mucosal epithelium. **f** PCA biplot of marker gene expression. Arrows represent gene loading vectors, colored by contribution magnitude (contribution 12–16; color bar). **g** Scree plot of condition indices for collinearity diagnostics. Horizontal dashed lines indicate thresholds for severe (red, >30) and moderate (orange, >15) multicollinearity.

Given the limitation of a small sample size of scRNA-seq and spatial transcriptome data, we established a large clinical cohort (*n* = 116) with a 1-year follow-up (Fig. 4a). The clinical cohort consisted of 94 NREOLP and 22 REOLP, with an average age of 41.4 years. Epidemiological analysis revealed no significant differences in sociodemographic characteristics during the inflammatory development of oral mucosal epithelium (Table S1).

To comprehensively capture the immunological characteristics of the inflammatory development in the oral mucosal epithelium, the analysis of cell type fraction and single-sample gene set enrichment analysis (ssGSEA) revealed the fraction of pyroptotic epithelial cells, TREM1^+^ Mφ, and pathogenic Th17 cells, along with the corresponding signaling pathways were up-regulated during the inflammatory development of oral mucosal epithelium (Fig. 4b). The expression of markers in pyroptotic epithelial cells (GSDMD, HMGB1), TREM1^+^ Mφ (CD68, TREM1, and IL1B), and pathogenic Th17 cells (IL-17A, CSF2) significantly increased, and exhibited stronger correlations during the inflammatory development of oral mucosal epithelium while the expression of KRT14 remained significantly unchanged (Fig. 4c, d).

Univariate logistic regression analysis identified that the aforementioned cell markers markedly increased the risk of the inflammatory development of oral mucosal epithelium (Fig. 4e), while multivariable logistic regression analysis confirmed IL1B as the sole independent risk factor (odds ratio [OR] = 2.38, *p* = 0.020; Table S2). Despite acceptable variance inflation factors (VIFs: <3), principal component analysis (PCA) biplot (Dim1 = 54.8%, Dim2 = 14.1%; Fig. 4f) and collinearity diagnostics (condition index: 18.54–105.65; Fig. 4g and Table S3) revealed the coordinated expression patterns between pyroptotic epithelial cells, TREM1^+^ Mφ, and pathogenic Th17 cells-linked genes, reflecting their mechanistic synergy in driving oral mucosal inflammation, which likely explain the loss of individual significance in multivariable analysis.

To characterize marker-gene associations while accounting for collinearity and validate result robustness, we performed ridge regression and bootstrap resampling. In the ridge regression model, all marker genes exhibited positive coefficients (range: 0.032–0.066), with 95% CI not overlapping zero-indicating consistent positive associations with the development of oral mucosal inflammation (Table S4). Bootstrap validation further supported the stability of these associations (Table S4). Additionally, the case-control imbalance of the clinical cohort (*n* = 22 REOLP vs. 94 NREOLP) may further constrain statistical power to resolve pathway interactions, as evidenced by the moderate contribution of key gene clusters (12–16% in PCA; Fig. 4f).

### Cytological experiments confirmed epithelial pyroptosis-induced TREM1^+^ Mφ promoted pathogenic Th17 cell differentiation via IL-1β

**Fig. 5:**
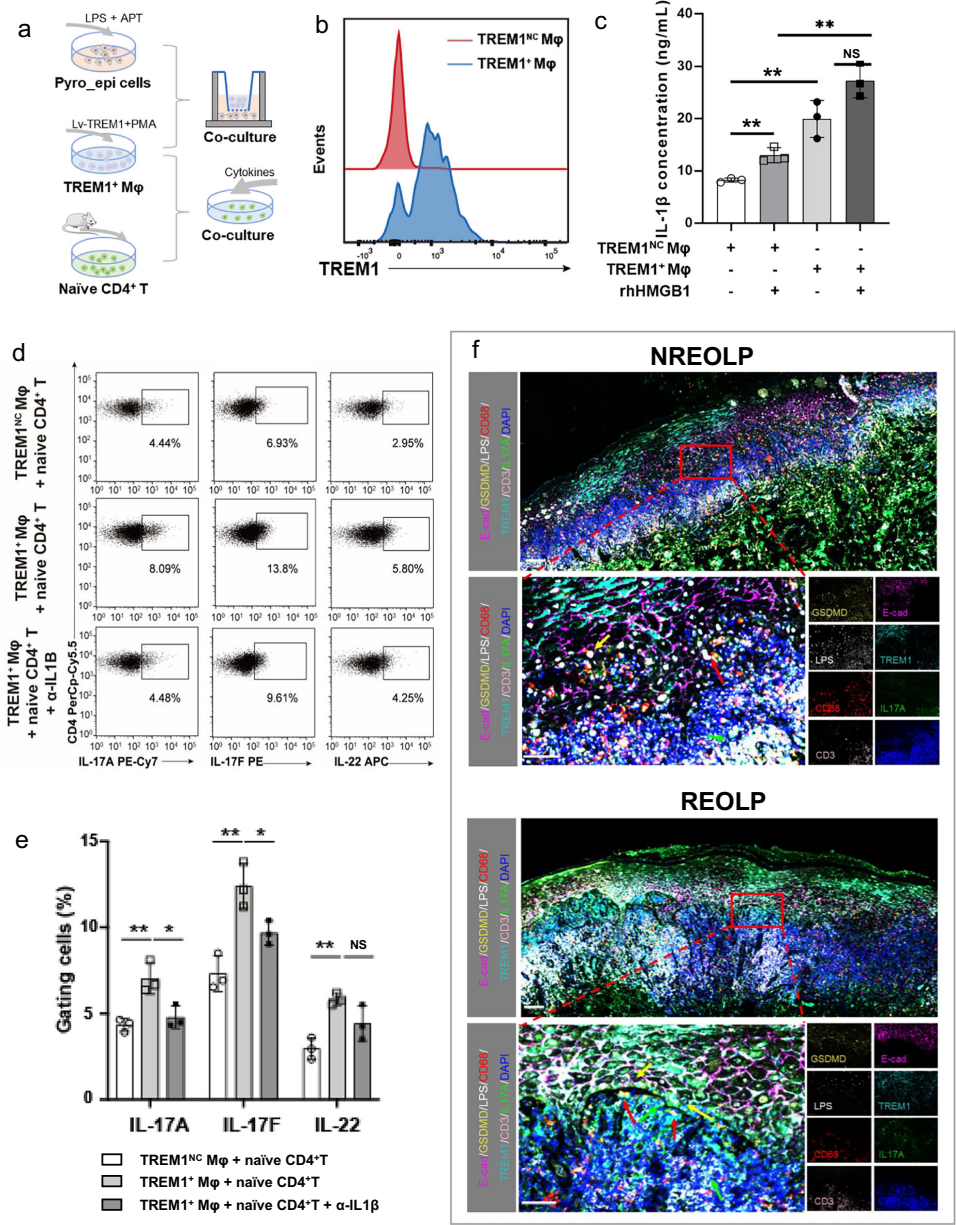
Cytological experiments and mIHC confirmed that TREM1^+^ Mφ promoted pathogenic Th17 cell differentiation triggered by epithelial pyroptosis. **a** Flow chart of cytological experiments. **b** Flow cytometry showing the TREM1 expression of the TREM1^+^ Mφ and TREM1^NC^ Mφ. **c** Elisa assay depicting the concentration of IL-1β in TREM1^+^ Mφ and TREM1^NC^ Mφ with/without the stimulation of recombinant human HMGB1 (rhHMGB1), respectively. Note: **p < 0.01, NS not statistically significant. Flow cytometry (**d**) and statistical analysis (**e**) of the c**e**ll proportion of CD4^+^ IL-17A^+^ cells, CD4^+^ IL-17F^+^ cells, and CD4^+^ IL-22^+^ cells after murine naïve CD4^+^ T cells co^-^cultured with the supernatants of TREM1^+^ Mφ, IL-6 and IL-23, with/without neutralizing anti-IL-1β (α-IL-1β). Note: *p < 0.05, **p < 0.01, NS not statistically significant. **f** Representative images of mIHC in NREOLP (top) and REOLP (bottom). Scale bars: 100 µM (top) and 50 µM (bottom). Yellow arrows pointed to LPS^+^ GSDMD^+^ E-cad^+^ cells. Red arrows pointed to TREM1^+^ CD68^+^ cells. Green arrows pointed to IL-17A^+^ CD3^+^ cells.

Next, we sought protein-level evidence to confirm that epithelial pyroptosis-induced TREM1^+^ Mφ could activate pathogenic Th17 cells to exacerbate oral mucosal inflammation (Fig. 5a). Firstly, we established a pyroptotic epithelial cell model using lipopolysaccharide (LPS) and adenosine 5’-triphosphate (ATP) [21]. The protein expression of N-terminal domain GSDMD (NT-GSDMD) and the HMGB1 concentration of the supernatants (*p* < 0.001) were significantly elevated in the pyroptotic epithelial cell model (Fig. S3a, b), indicating the successful establishment of the pyroptotic epithelial cell model. We then co-cultured pyroptotic epithelial cells and THP-1 cells with/without 5 μg/ml neutralizing anti-HMGB1 (α-HMGB1), and the results showed that pyroptotic epithelial cells could induce THP-1 cells to differentiate into TREM1^+^ Mφ (*p* = 0.004) through HMGB1 (*p* = 0.006; Fig. S3c, d).

Concurrently, we utilized TREM1 overexpressed lentivirus to establish the TREM1^+^ Mφ model using the THP-1 cell line (Fig. 5b and Fig. S3e). We observed that TREM1^+^ Mφ secreted high levels of IL-1β (*p* = 0.005; Fig. 5c), consistent with previous findings [14]. Additionally, TREM1^+^ Mφ secreted more IL-1β when co-cultured with 5 μg/mL recombinant human HMGB1 (rhHMGB1; *p* = 0.057; Fig. 5c) or pyroptotic epithelial cells (*p* < 0.001; Fig. S3f).

Furthermore, TREM1^+^ Mφ supernatants were co-cultured with murine naïve CD4^+^ cells to explore the TREM1^+^ Mφ regulation of Th17 cells under different cytokine stimulations. Flow cytometry results showed that in the presence of IL-6 and TGFβ (a condition for generating non-pathogenic Th17 cells [22]), the cell proportion of CD4^+^ IL-17A^+^, CD4^+^ IL-17F^+^, and CD4^+^ IL-22^+^ cells was not enhanced in the group of naïve CD4^+^ cells treated with TREM1^+^ Mφ supernatants (Fig. S3g, h), indicating that TREM1^+^ Mφ did not affect the differentiation of non-pathogenic Th17 cells. Conversely, under IL-6 and IL-23 stimulation (a condition for generating pathogenic Th17 cells [22]), the proportion of CD4^+^ IL-17A^+^ cells (*p* = 0.009), CD4^+^ IL-17F^+^ cells (*p* = 0.006), and CD4^+^ IL-22^+^ cells (*p* = 0.002) was markedly increased in the group of naïve CD4^+^ T cells treated with TREM1^+^ Mφ supernatants and significantly decreased (CD4^+^ IL-17A^+^ cells *p* = 0.026, CD4^+^ IL-17F^+^ cells *p* = 0.034, and CD4^+^ IL-22^+^ cells *p* = 0.086) when co-stimulated with 5 μg/mL neutralizing anti-IL-1β (α-IL-1β; Fig. 5d, e), indicating that TREM1^+^ Mφ could drive the differentiation of pathogenic Th17 cells via IL-1β.

These findings confirm that TREM1^+^ Mφ, activated by HMGB1 from epithelial pyroptosis, stimulates pathogenic Th17 cells to secrete pro-inflammatory cytokines, including IL-17A, IL-17F, and IL-22, via IL-1β.

### MIHC unveiled the spatial dynamic changes of TREM1^+^ Mφ, epithelial pyroptosis, and Th17 cells in the inflammatory development of oral mucosal epithelium

We utilized mIHC and IHC to explore the spatial location and potential intercellular interactions of TREM1^+^ Mφ, Th17 cells, and pyroptotic epithelial cells during inflammation of the oral mucosal epithelium. The results of mIHC revealed that pyroptotic epithelial cells (GSDMD^+^ E-cad^+^ cells; *p* = 0.043) were closer with TREM1^+^ Mφ (TREM1^+^ CD68^+^ cells; *p* < 0.001) and Th17 cells (IL-17A^+^ CD3^+^ cells; *p* = 0.008), accompanied by their elevated proportion during the inflammatory development of oral mucosal epithelium (Fig. 5f and Fig. S4a–c). Notably, LPS was co-located with partial pyroptotic epithelial cells (LPS^+^ GSDMD^+^ E-cad^+^ cells), and the cell proportion increased (*p* = 0.013) with the inflammatory development of oral mucosal epithelium (Fig. 5f and Fig. S4d), repminding an association between LPS and epithelial cell pyroptosis during the inflammatory development of oral mucosal epithelium. In addition, the protein expression of HMGB1 also increased in the epithelium during the inflammatory development of oral mucosal epithelium (*p* = 0.024; Fig. S4e). These confirm the potential spatial intercellular communication among TREM1^+^ Mφ, Th17 cells, and pyroptotic epithelial cells.

### ORGUAMIA exhibited TREM1^+^ Mφ participated in the inflammatory development of chronic digestive disorders based on public datasets

**Fig. 6:**
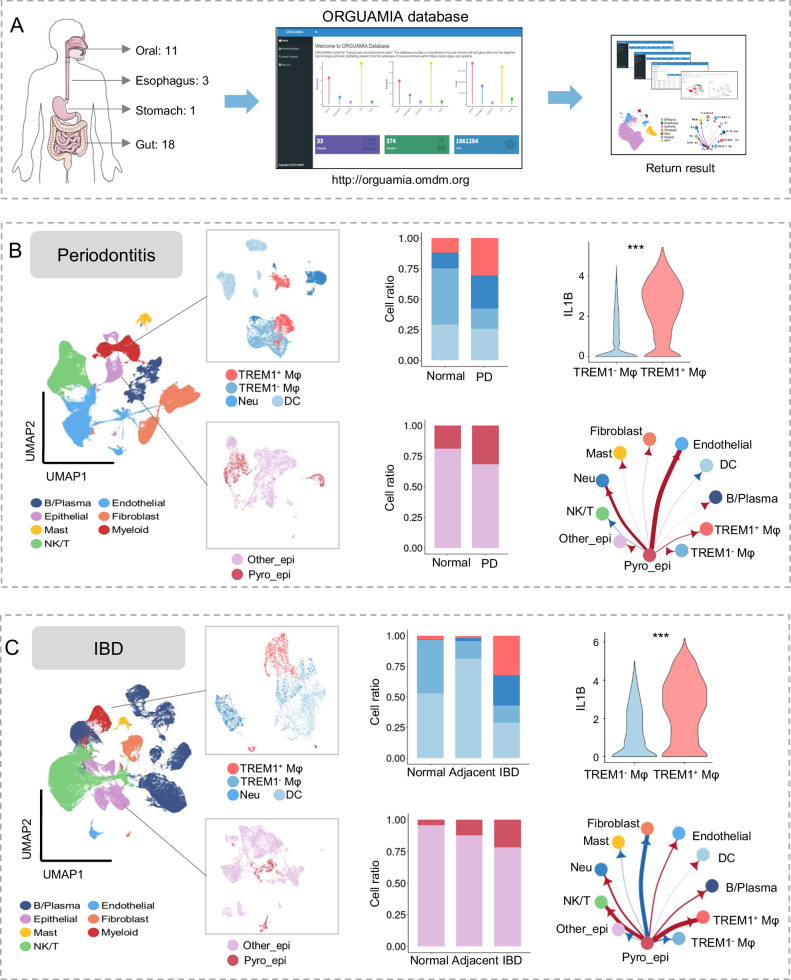
ORGUAMIA database uncovered that TREM1^+^ Mφ participated in the inflammatory development of chronic digestive disorders. **A** Schematic of the establishment of the ORGUAMIA database. The role of epithelial pyroptosis-induced TREM1^+^ Mφ in periodontitis (PD) datasets (**B**) and IBD datasets (**C**). Left, UMAP plot of cell colors by cell types, myeloid cell types, and epithelial cell types. Middle, Histogram showing the cell proportions of myeloid cell types (top) and epithelial cell types with disease subtypes (bottom). Top right, violin plot showing the IL1B expression in Mφ subtype. Bottom right, circle plot of the intercellular communication from pyroptotic epithelial cells to other cells. Line width, the intercellular communication strength. Red arrow, the intercellular communication strength in PD/IBD was higher than in normal. Blue arrow, the intercellular communication strength in normal was higher than in PD/IBD.

To explore the crucial role of TREM1^+^ Mφ in chronic digestive diseases, we collected 33 related datasets of scRNA-seq or spatial transcriptome from the Gene Expression Omnibus (GEO) database (Table S5). Subsequently, we established ORGUAMIA (https://orguamia.omdm.org), a spatiotemporal interactional online database based on shiny (Fig. 6a).

Of those, we integrated 12 scRNA-seq datasets to explore the role of TREM1^+^ Mφ in chronic digestive disorders. The results revealed an increased proportion of TREM1^+^ Mφ, characterized by the high IL1B expression, in periodontitis, eosinophilic esophagitis (EoE), IBD, and perianal fistulizing Crohn’s disease (Perianal-CD) (Fig. 6b, c, and Fig. S5a–c). Moreover, the cell proportion of pyroptotic epithelial cells was elevated in periodontitis, EoE, IBD, and perianal-CD, with heightened intercellular communication with TREM1^+^ Mφ, except in EoE (Fig. 6b, c and Fig. S5a, b). Whereas the cell proportion of Mφ in chronic atrophic gastritis (CAG) was lower than that in non-atrophic gastritis (NAG). TREM1^+^ Mφ was not found in CAG or NAG (Fig. S5d), which might be caused by the unique physiological environment of the stomach. Under the strong acid condition, microbes were less in the stomach and produced less LPS in the stomach compared with oral or intestinal mucosa, which may explain that TREM1^+^ Mφ was not found in CAG or NAG in our analysis.

These findings underscore the pivotal pro-inflammatory role of TREM1^+^ Mφ in chronic digestive tract disorders, potentially triggered by pyroptotic epithelial cells, especially in the oral and intestinal mucosa.

## Discussion

Overall, this study demonstrates that epithelial pyroptosis-induced TREM1^+^ Mφ can activate pathogenic Th17 cells to exacerbate oral inflammation and play an extensive pro-inflammatory role in chronic digestive tract disorders. Moreover, ORGUAMIA, the powerful online database of the oral-gut immune atlas, allows researchers to access and utilize scRNA-seq or spatial transcriptome data without technological barriers.

Pyroptosis, a gasdermin-mediated regulated cell death, can recruit immune cells and expand the immune cascade [23, 24]. GSDMD is an essential protein of pyroptosis, which is cleaved as NT-GSDMD and C-terminal domain GSDMD [25, 26]. NT-GSDMD migrates onto the plasmalemma and eventually forms transmembrane pores, allowing the release of ions and small molecular weight proteins such as IL-1β and IL-18 [26, 27]. After the cell membrane ruptured, cellular contents, such as HMGB1 and lactate dehydrogenase, were released to exacerbate the inflammatory response [23, 24]. Tan et al. [13] found that intestinal epithelial pyroptosis recruits Mφ and neutrophils by releasing HMGB1 to participate in the pathogenesis of intestinal inflammation. In this study, we observed that epithelial pyroptosis contributes to oral mucosal inflammation and induces TREM1^+^ Mφ to secrete massive IL-1β to accelerate epithelial inflammation of the oral mucosa, which accordance with previous studies [28, 29]. Furthermore, epithelial pyroptosis-induced TREM1^+^ Mφ played a pivotal role in chronic digestive tract disorders. These findings provide new insight into the common mechanism of other chronic inflammatory diseases with similar pathogenesis.

In addition, we observed the co-localization of LPS with pyroptotic epithelial cells, particularly in the basal epithelial layer, with the increased cell proportion during the inflammatory development of oral mucosal epithelium. Previous studies suggested that LPS can activate GSDMD through CASP4/5/11 to induce pyroptosis [30, 31]. Thus, we speculated that LPS may induce epithelial pyroptosis to promote the inflammatory development of oral mucosal epithelium, though additional evidence is required for confirmation.

After activation through epithelial pyroptosis, TREM1^+^ Mφ induced pathogenic Th17 cells via IL-1β to exacerbate oral mucosal inflammation. Pathogenic Th17 cell, as a special Th17 cell subset, triggered and aggravated the immune response of chronic inflammatory diseases, such as periodontitis, IBD, and rheumatoid arthritis, through IL-17, IL-21, IL-22, IL-23, and GM-CSF (gene: CSF2) [5, 32–34]. IL-17 and IL-21 induced intestinal epithelial cells to secrete C-C chemokine motif ligand 20 (CCL20) and IL-8, thereby recruiting immune cells to intensify inflammatory response. Additionally, IL-17 and IL-21 can induce myofibroblasts to secrete matrix metalloproteinases to finally damage the epithelial barrier [20, 35]. Li et al. [36] demonstrated that IL-17A induces nasal epithelial cell pyroptosis. A recent meta-analysis showed that Th17 cells are involved in the inflammatory development of OLP [37]. Therefore, we will further elucidate the effect of pathogenic Th17 cells on epithelial cells during oral mucosal inflammation.

The rapid development of high-throughput sequencing technology has greatly advanced the progress of medical research, albeit posing challenges for researchers without a bioinformatics background. To harness the full medical potential of these high-throughput data, we established ORGUAMIA, a publicly-available online database based on web construction, using scRNA-seq and spatial transcriptome datasets of chronic digestive disorders. ORGUAMIA revealed the pivotal role of epithelial pyroptosis-induced TREM1^+^ Mφ in chronic digestive tract disorders and aids researchers in exploring other potential mechanisms of chronic digestive disorders.

This study had some limitations. Firstly, there is no currently accepted animal model for OLP research, we did not use a mouse model of OLP. Secondly, the underlying reason for epithelial pyroptosis and the effect of pathogenic Th17 cells on epithelial cells need to be clarified. Finally, the appearance and function of ORGUAMIA still need improvement.

In summary, this study demonstrated epithelial pyroptosis-induced TREM1^+^ Mφ drive pathogenic Th17 cells via IL-1β, intensifying oral mucosal inflammation and exhibiting a broad pro-inflammatory role in chronic digestive tract orders, implying TREM1^+^ Mφ might be a promising target for mitigating chronic digestive inflammation. Furthermore, ORGUAMIA is a publicly available, efficient, and convenient tool for researchers to investigate oral-gut immune interactions using scRNA-seq or spatial transcriptome data without technological barriers.

## Materials and methods

### Patient recruitment and mice model establishment

The participants were recruited from the Department of Oral Medicine of West China Hospital of Stomatology between 2019 and 2022. Participants diagnosed with OLP based on clinical symptoms and pathology underwent a 1-year follow-up, either through in-person visits or telephone consultations. All patients provided informed consent and were categorized into two groups based on the number of erosive events observed during the follow-up period: NREOLP (≤3 times) and REOLP (>3 times). All procedures were conducted in accordance with the Declaration of Helsinki and approved by the Ethics Committee of West China Hospital of Stomatology Sichuan University (WCHSIRB-D-2017-021).

C57BL/6 mice were housed at 25 °C with access to standard chow and water. Naïve CD4^+^ T cells were isolated from the spleens and lymph nodes of sacrificed C57BL/6 mice using cell strainers. The protocols were approved by the Ethics Committee of West China Hospital of Stomatology Sichuan University (WCHSIRB-D-2023-620).

### Sample preparation and library establishment of scRNA-seq

Thirteen oral mucosal samples (normal oral mucosa = 3, NREOLP = 4, REOLP = 6) were washed with D-PBS twice and cut into fragments with a diameter of approximately 1–2 mm. These fragments were then immersed in a mixture of human Whole Skin Dissociation Kit (Miltenyi) for infiltration. The mixture was digested in a water bath at 37 °C for 2.5 h and dissociated by a gentleMACS^TM^ Octo Dissociator for 15 s every 0.5–1 h. Subsequently, the mixture was filtered using 70 μm MACS Smartstrainers (Miltenyi), followed by the addition of 1 mL DMEM to terminate digestion and centrifugation at 4 °C and 400 rcf for 15 minutes (min). The cell precipitates were then subjected to red blood cell lysis using Red Blood Cell Lysis Buffer (Abcam), and dead cells were removed using Easysep^TM^ Dead Cell Removal (Annexin V) Kit (STEMCELL). Finally, the single-cell suspension was resuspended in D-PBS and counted using trypan blue.

The library establishment method followed previously described procedures [38]. Briefly, the single-cell suspension was combined with barcoded beads to produce single-cell Gel Beads-in-Emulsion (GEMs) using the Chromium Single Cell 3’GEM Kit v3 (10× GENOMICS; #PN-1000077) on the 10×chromium platform. GEMs were dissolved, cells were lysed to release mRNA, and cDNAs were amplified. The library was constructed using the Chromium Single Cell Library Kit v3 (10× GENOMICS; #PN-1000078) and the Chromium Single Cell 3’GEM Gel Bead Kit v3 (10× GENOMICS; #PN-1000076) and sequenced on an Illumina NextSeq 500 System.

### Spatial transcriptome sample preparation and data acquisition

Six oral mucosal samples (3 NREOLP and 3 REOLP) were frozen and embedded in dry ice. Frozen tissues were sectioned (10 μm) and attached to ST microarrays (https://www.10xgenomics.com/). First, slides were dehydrated, stained with Hematoxylin and Eosin, and imaged using a 3D HISTECH Pannoramic MIDI FL scanner. Subsequently, slides were permeated to obtain fluorescent-labeled cDNA. The cDNA was digested and fragmented to construct the DNA library. The DNA library was sequenced using the Illumina novaseq 6000 system.

### Sample preparation of clinical follow-up cohort

A total of 116 clinical samples were collected and stored at −80 °C. All samples were extracted from total RNA to construct the sequencing library using the NEBNext Ultra^TM^ RNA Library Prep Kit for Illumina (NEB; #E7770) and finally sequenced using the Illumina Hiseq X Ten and Illumina novaseq 6000.

### ScRNA-seq analysis

#### Cell clustering and annotation in scRNA-seq

Seurat (v4.3.0) package was the major tool used for scRNA-seq analysis [39]. Features detected in less than 3 cells, cells detected in less than 200 features, and cells detected with more than 15% mitochondrial content were filtered out. The integrated matrix was normalized using the “sctransform v2” function and clustered using the “FindClusters” function. Cell annotation was labeled using classical cell markers. The results of cell clustering and annotation were exhibited using the Uniform Manifold Approximation and Projection (UMAP) plot and dot plot.

#### Cell composition analysis

To understand the composition of different diseases and cell subtypes, we utilized the “prop. Table” function of R (v4.1.3) to perform cell composition analysis and visualize by the piled histograms.

#### DAseq analysis

DAseq is an algorithm for detecting differentially abundant cell subpopulations to discriminate biological states in scRNA-seq data [19]. DA cell subpopulations were identified using the DA-seq algorithm through multiscale neighborhood scoring, logistic regression-based DA measure integration, gene expression clustering of cells with significant abundance differences, and validation via marker gene selection using stochastic gates and differential expression analysis [19]. We utilized the DAseq package to find the DA cells using the “getDAcells” function ( | log_2_ fold change [FC]| > 0 and FDR < 0.05) and identify the DA regions using the “getDAregion” function. The markers of DA regions were selected using the “STGmarkerFinder” function. The results are shown in the UMAP plot.

#### Intercellular communication analysis

The cellchat package was used to perform cell-cell communication analysis [40]. We utilized “computeCommunProb” function to compute the intercellular communication probability. The intercellular communication probability was inferred based on the CellChatDB database combined with a PPI network. The minimum number of cells required in each cell group for intercellular communication was set to 10 with the default parameter. We visualized the intercellular communication probability between two groups through the “netAnalysis_singnalingRole_scatter” function and the “netVisual_diffInteraction” function.

#### Pseudotime trajectory analysis

The monocle package was used to construct pseudotime trajectory and detect the pseudotime trajectory of cell subtypes. Highly variable genes were selected using the “dispersionTable” function from genes detected in >=10 cells. Next, the data underwent dimension reduction using the DDRTree method, and pseudotime trajectory construction was achieved by ordering cells using the “orderCells” function.

#### Gene Set Variation Analysis (GSVA)

To avoid missing biologically important genes without differential expression, GSVA analysis combined with c5.all.v7.5.1.symbols file and c2.all.v7.5.1.symbols file (http://www.gsea-msigdb.org/gsea/downloads.jsp) was performed using the “gsva” function of the GSVA package [50]. Next, differential GSVA was performed using the limma package and adjusted using the Benjamini−Hochberg method [41]. The differential pathways were selected using |log_2_ FC | > 0 and FDR < 0.05.

#### Correlation analysis

The correlation of pathways was calculated using the Pearson method and visualized using the “dittoScatterHex” function of the dittoSeq package and the “ggpairs” function of the GGally package. A correlation coefficient >0.4 and *p* < 0.05 were deemed as high correlations.

#### Differential analysis

RNA expression of different groups was tested using the *t*-test and shown in the violin plot.

#### Transcription factor analysis

The SCENIC package was used to select the active transcription factors and the following downstream regulatory gene in TREM1^+^ Mφ [42]. All parameters were set to default and the details of SCENIC are available online at https://github.com/aertslab/SCENIC.

#### PPI

This study utilized the “Multiple proteins” model of the STRING database to perform PPI analysis (https://cn.string-db.org/).

### Spatial transcriptomic analysis

#### Cell clustering and annotation in the spatial transcriptome

The Seurat and Giotto packages were the major tools used for spatial transcriptome analysis [39, 43]. The expression matrix was normalized using the NormalizeData function and scaled using the “ScaleData” function with 2000 high-variable genes. The expression matrix was transformed using the “SCTransform” function to reduce the dimension with PCA and UMAP. Cell clustering was combined with the “FindClusters” function of the Seurat package and the “doLeidenSubCluster” function of the Giotto package. Cell annotations were performed using marker genes and deconvolved with the scRNA-seq data described above using the “runRankEnrich” function. The cell annotations were visualized using the “plotMetaDataCellsHeatmap” function.

#### Spatial intercellular communication analysis

CellChat v2 is an updated version of Cellchat, which performs spatial intercellular communication analysis [44]. The procedure is the same as described above. The minimum number of spots required in each group for intercellular communication was set to 1 with the default parameter. We visualized the intercellular communication probability between two groups using the “netVisual_spatial” function.

### Analysis in the clinical follow-up cohort

#### Data pretreatment of clinical follow-up cohort

After the raw data, the count data was transformed into TPM data using the IOBR package [45]. To reduce the batch effect, the log_2_(TPM + 1) data were corrected using the “ComBat” function of the sva package [46].

#### Cell type fraction based on deconvolution

Bayes Prism was used to perform cell-type deconvolution for bulk RNA-seq using scRNA-seq data [47]. This study employed the BayesPrism package to investigate the cell-type fraction of bulk RNA-seq data. Protein-coding genes were selected using the “select.gene.type” function, followed by BayesPrism analysis using the “run.prism” function.

#### ssGSEA analysis

To clarify the immune changes during the inflammatory development of oral mucosal epithelium, this study utilized ssGSEA analysis with the c5.all.v7.5.1.symbols file (http://www.gsea-msigdb.org/gsea/downloads.jsp) through the “ssgsea” algorithm of the GSVA package. In addition, we combined epithelial markers (KRT6A, KRT10, KRT14, KRT19, and CDH1) with pyroptosis-related markers described in a previous study [48] to create a “Pyroptosis in epithelial cell” signaling pathway. Next, the ssGSEA underwent the same differential analysis procedure as GSVA.

#### Univariate and multivariable logistic regression analyses

Univariate logistic regression analysis was conducted to assess associations between clinical characteristics/selected biomarkers (TREM1^+^ Mφ, pyroptotic epithelial cells, and pathogenic Th17 cells) and outcomes using the “glm” function in the stats package, with results presented in forest plots. Multivariable logistic regression was performed on significant gene markers using the same package, adjusting for clinical characteristics. Multicollinearity was evaluated through VIFs calculated by the perturb package’s “vif” function, with additional diagnostic verification using condition indices and variance decomposition from its “colldiag” function.

#### Ridge regression model and bootstrap resampling

Ridge regression effectively mitigates multicollinearity via L2 regularization and counteracts overfitting in small samples through coefficient shrinkage, thereby enhancing estimator stability. The ridge regression analysis was conducted using the “cv.glmnet” function from the glmnet package. Bootstrap resampling (1000 iterations) was performed with the “boot” function in the boot package to verify coefficient stability.

### ORGUAMIA establishment and analysis

#### ORGUAMIA establishment

ORGUAMIA, a spatiotemporal interaction-enabled online database, integrates 33 publicly available single-cell/spatial transcriptomics datasets curated from the NCBI GEO repository (https://www.ncbi.nlm.nih.gov/geo/). This platform was developed using the Shiny framework (v1.10.0), providing real-time analysis of cellular dynamics. The interactive visualization interface is powered by the bslib package (v0.9.0), enabling dynamic theme customization. Furthermore, the user interface was optimized with the shinydashboard toolkit (v0.7.3) to deliver modular dashboards for multi-omics exploration.

#### Data pre-treatment and analysis

All datasets were processed by uniformly normalizing with the “SCTransform” function of Seurat (v4.3.0) and correcting batch effects using the “RunHarmony” function of harmony (v1.2.0). Downstream analyses followed the standardized Seurat (v4.3.0) workflow. We further selected and integrated 12 datasets to explore the role of epithelial pyroptosis-induced TREM1^+^ Mφ, with patient numbers ≥5 and cell types included all cell types or at least all immune cells. The 12 scRNA-seq datasets included periodontitis datasets (GSE164241 [11] and GSE171213 [49]), EoE datasets (GSE201153 [50] and GSE218607 [51]), gastritis datasets (GSE134520 [52]), IBD datasets covering all cells (GSE214695 [53], GSE231993 [54], GSE182270 [55], and GSE150115 [56]), IBD datasets only including immune cells (GSE125527 [55, 57] and GSE162335 [58, 59]), and perianal fistulizing Crohn’s disease datasets (GSE225199 [59]).

### Cytological experiments

#### Cell culture and cell model establishment

HOK cell line, a human oral epithelial cell line, was cultured in a defined keratinocyte serum-free medium (Gibco, USA, #10744019). THP-1, a human monocyte cell line, was cultured in 1640 medium with 10% serum and 1% penicillin-streptomycin. All cell lines were recently authenticated (Files S1 and S2) and maintained at 37 °C with 5% CO_2_. Murine naïve CD4^+^ cells were obtained from mouse spleen and lymph nodes through grinding and passed through a 70 μM filter membrane (Biosharp, China, #23116232), lysed using red cell lysis buffer (Biosharp, China, #BL503B), and sorted using magnetic beads of CD62L^+^ CD4^+^ T cells (Miltenyi Biotec, German, #130-106-643).

To establish a cell model of pyroptosis in oral epithelial cells, we first utilized 3 μg/mL LPS (Sigma, #L3129) to induce the HOK cell line for 12 h when cell confluency reached 80% and then utilized 3 mmol/L ATP (Sigma, # 11140965001) to further stimulate the HOK cell line for 4 h. The control group used the same amount of PBS to simultaneously stimulate the HOK cell line.

To obtain a TREM1^+^ Mφ cell model, a THP-1 cell line with 70% cell density was first infected with TREM1 overexpressed lentivirus (Genechem Co., LTD, China) for 48 h. The control group was the THP-1 cell line with lentivirus delivering the empty vector. Next, we utilized 0.1 μg/mL PMA (Sigma, #P8139) to stimulate the THP-1 cell line with TREM1 overexpression or empty vector for 3 days.

To induce Th17 cells, murine naïve CD4^+^ T cells (2 × 10^5^ cells/well) were suspended in 1.5 μg/mL anti-CD28 containing T cell media and co-cultured with 500 mL supernatants of TREM1^+^ Mφ model and stimulated with 5 μL IL-6 + 5 μL TGFβ (non-pathogenic Th17 cells) or 5 μL IL-6 + 5 μL IL-23 (pathogenic Th17 cells) in anti-CD3 coated culture plates for 3 days.

#### Western blotting

Protein was extracted through lysis in RIPA buffer and measured using the bicinchoninic acid method. Other procedures have been described in a previous study [28]. The primary antibodies included GSDMD (1:1000; Bioss, China, #BS-14287R), TREM1 (1:600; Proteintech, China, #11791-1-AP), and β-Actin (1:2000; Cell Signaling, U.S., #4970S). The images were captured using ECL (EMD Millipore, WBKLS0500).

#### Elisa assay

HMGB1 (Jianglaibio, China; JL13693) and IL-1β (Jianglaibio, China; JL136620) were used to perform enzyme-linked immunosorbent assay (ELISA). The detailed experimental procedures are described in the manufacturer’s instructions manual. Experiments were repeated three times.

#### Flow cytometry

To assess the establishment of the TREM1^+^ Mφ model, PMA-induced THP-1 cells infected with TREM1 lentivirus were surface stained with an antibody against TREM1 (1:200; Proteintech, China, #11791-1-AP) for 30 min. To isolate Th17 cells post-stimulation of cytokines and the TREM1^+^ Mφ model following naïve CD4^+^ T cell sorting, cells were pretreated with Cytofix/Cytoperm^TM^ buffer (BD Biosciences, U.S., #1361481) for 20 min and stained with antibodies against IL-17A (1:100, Invitrogen, #25-7177-82), IL-17F (1:100, Invitrogen, #12-7471-82), and IL-22 (1:100, Invitrogen, #17-7222-82) for 40 min. Cells were detected using LSRFortessa (BD Biosciences) and analyzed using FlowJo software (V10.4).

#### Cell co-culture assays

This study used six-well plates and a 0.4 μm Transwell chamber for the cell co-culture assay. TREM1^+^ Mφ (5 × 10^5^ cell/well) were seeded in the upper chamber and pyroptotic HOK cells (5 × 10^5^ cell/well) were seeded in the lower chamber for 24 h. Next, we collected the supernatants to determine the IL-1β concentration.

### MIHC and IHC

#### MIHC

Six tissue samples (NREOLP = 3, REOLP = 3) were used for mIHC. Paraffin sections were pretreated by dewaxing, rehydration, and antigen retrieval. Subsequently, sections were subjected to sequential staining cycles using the IRISKit HyperView mIF kit (LuminIris, China; #MH010101). The primary antibodies included were GSDMD (1:500; Bioss, China; #BS-14287R), E-cadherin (1:1000; HUABIO, China; #ET1607-75), LPS (1:100; Cloud-clone, China; MAB526Ge22), TREM1 (1:1000; Proteintech, China; #11791-1-AP), CD68 (1:200; Abcam, England; #ab201340), IL-17A (1:300; Proteintech, China; #26163-AP), CD3 (1:1000; HUABIO, China; #HA720082), and DAPI (1:500; Beyotime, China; #C1002). The secondary antibodies used include Rabbit poly-HRP (HUABIO, China; #HA1119) and Mouse poly-HRP (HUABIO, China; #HA1120). Images were obtained using a slide scanner (Olympus, VS200), and image registration was performed using Image J1.53t.

#### IHC

Six tissue samples (NREOLP = 3, REOLP = 3) were taken for IHC staining. Paraffin sections were treated in the same way as the pretreatment of multiplex immunofluorescence. The primary antibody HMGB1 (1:200; Servicebio, China, #GB11103) was incubated overnight at 4 °C. On the second day, the sections were incubated with the secondary antibody, Goat anti-rabbit immunoglobulin G-horseradish peroxidase (1:200; Servicebio, China, #GB23303), at room temperature for 50 min and subsequently taken for DAB chromogenic reaction. Finally, images were obtained using a slide scanner (Leica, Aperio VERSA8).

#### Statistical analysis of mIHC and IHC

Images from mIHC and IHC were analyzed using Qupath 0.4.3 [60]. All images were randomly selected from three fields to detect cell counts. The selected fields were further classified as epithelial cell zones and lamina propria lymphocytic infiltration zones to calculate the cell percentages in the above zones. Statistical tests were performed using GraphPad Prism 8. An unpaired Student’s t-test was used when the assumptions of homogeneity of variance (assessed by F-test, *p* > 0.05) and normality of data (assessed by Shapiro-Wilk test, *p* > 0.05) were met. Welch’s t-test was used when homogeneity of variance was violated (F-test *p* < 0.05) but normality held (Shapiro-Wilk test *p* > 0.05). The Mann-Whitney U test was used for non-normally distributed data (Shapiro-Wilk test *p* < 0.05).

## Supplementary information

supplementary information

WB

ad.checklist

## Data Availability

Single-cell RNA-seq data, spatial transcriptome data, and bulk RNA-seq data have been deposited in the GEO database (GSE211630, GSE213345, GSE213346) and Genome Sequence Archive database (HRA002370). Some of the data supporting the findings of this study are not publicly available due to privacy protection. Reasonable requests for access to these data can be directed to the corresponding author.

## Code availability

All original code has been deposited at GitHub (https://github.com/chxhstatis/oral-lichen-planus) and is publicly available as of the date of publication.
