# Supplementary material for: Epithelial pyroptosis-induced TREM1+ macrophages activate Th17 cells to accelerate oral mucosal inflammation
Source: Cell Death Discov. 2025 Nov 29;12:26. doi: 10.1038/s41420-025-02853-7 (PMC12811386; doi:10.1038/s41420-025-02853-7)
Supplement: Supplementary file 1 — supplementary information [file 41420_2025_2853_MOESM1_ESM.pdf]

Supplementary information

Epithelial pyroptosis-induced TREM1$^+$ macrophages activate Th17 cells to accelerate oral mucosal inflammation

This PDF file includes:

Fig. S1 to Fig. S5

Table S1 to Table S5

File S1 to File S2

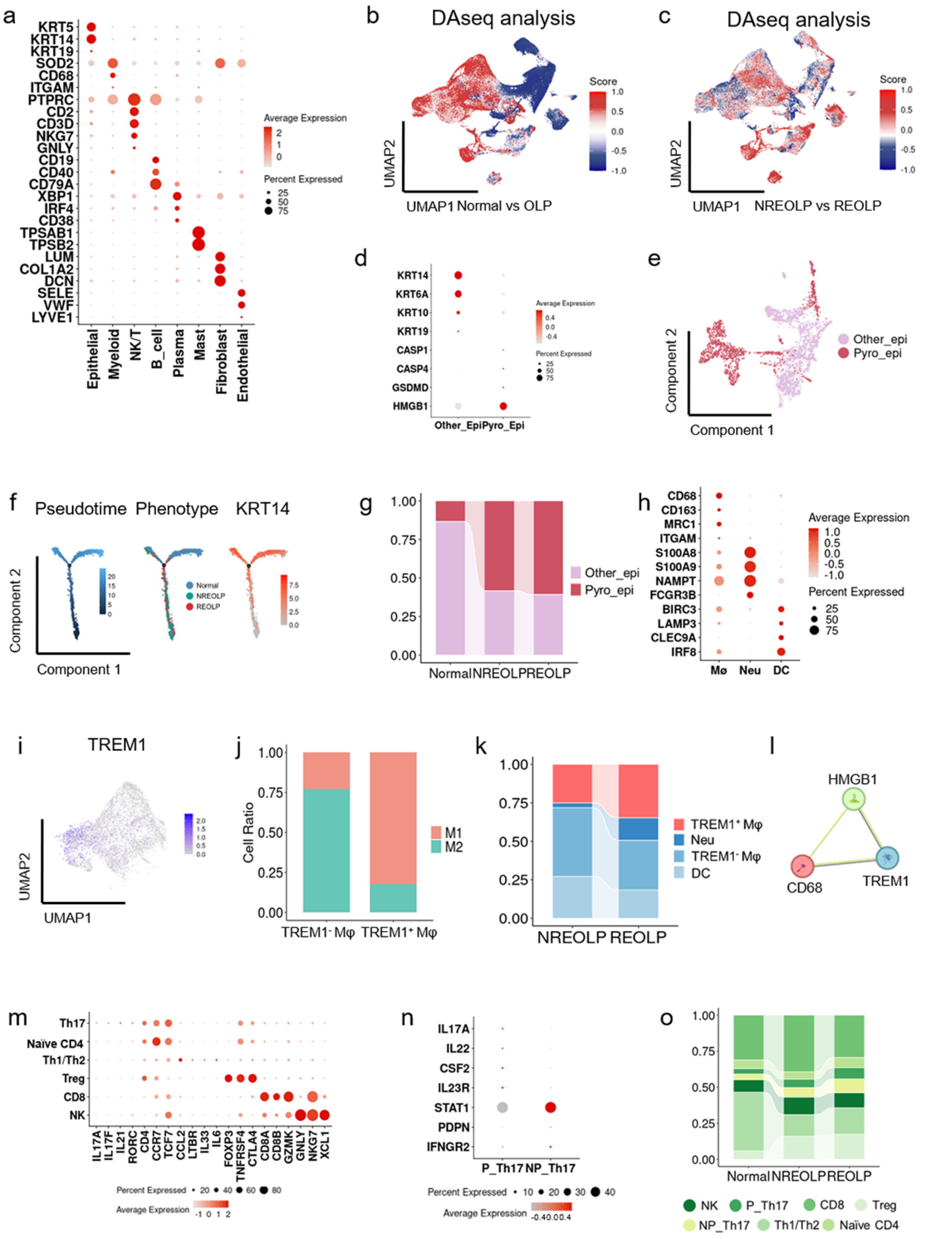

Fig. S1 Characteristics of cell types in the inflammatory development of oral mucosal epithelium. a, Dot plot displaying the marker genes of all cell types. Dot size, percentage of cells expressing marker gene; color scale, average marker gene expression. **b**, UMAP plot showing the differential abundance (DA) cells score in normal oral mucosa and OLP through DAseq analysis. Red: high DA score in OLP; Blue: high DA score in normal oral mucosa. **c**, UMAP plot showing the DA cells score in the inflammatory development of oral mucosal epithelium. Red: high DA score in REOLP; Blue: high DA score in NREOLP. **d**, Dot plot displaying the marker genes of epithelial cell types. Dot size, percentage of cells expressing marker gene; color scale,

average marker gene expression. **e**, UMAP plot of 3,215 cell colors by epithelial cell subtypes. **f**, Developmental trajectory of pseudotime (left), disease subtypes (middle), and KRT14 (right). **g**, Histogram showing the cell proportions of epithelial cells with disease subtypes. **h**, Dot plot displaying the marker genes of myeloid cell types. Dot size, percentage of cells expressing marker gene; color scale, average marker gene expression. **i**, UMAP plot showing TREM1 expression in Mφ. **j**, Histogram showing the cell proportions of M1/ M2 Mφ with TREM1^{+} Mφ/ TREM1^{-} Mφ. **k**, Histogram showing the cell proportions of myeloid cell types with disease subtype. **l**, PPI of HMGB1, CD68, and TREM1 based on STRING database. Yellow line: textmining. Grey line: co-expression. **m,n**, Dot plot displaying the marker genes of NK/T cell types (**m**) and Th17 cell types (**n**). Dot size, percentage of cells expressing marker gene; color scale, average marker gene expression. **o**, Histogram showing the cell proportions of NK/T cell types with disease subtype.

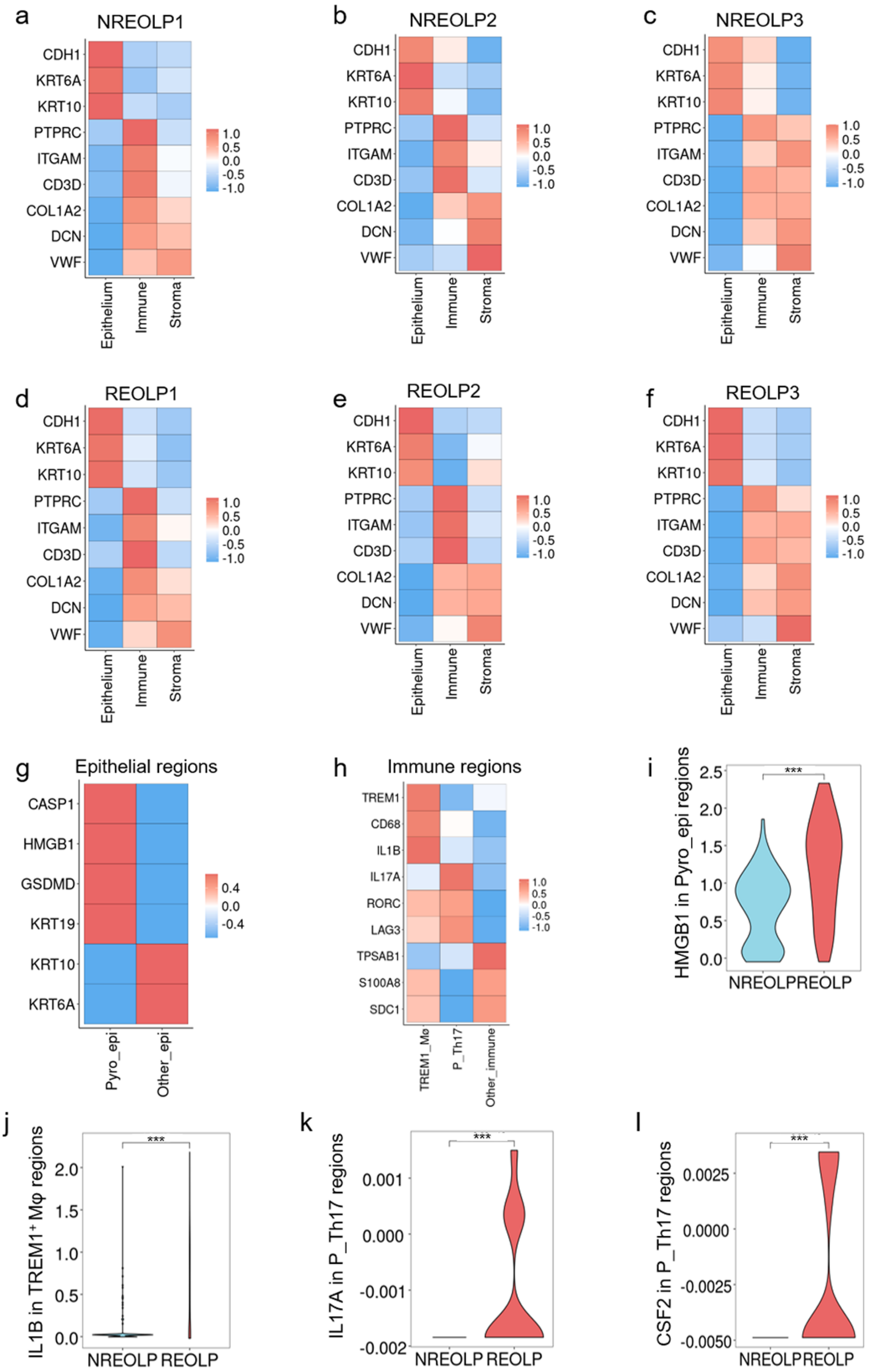

Fig. S2 Spatial characteristics of cell types in the inflammatory development of oral mucosal epithelium. a-f, Heatmap displaying the marker genes expression of

tissue regions in NREOLP1 (**a**), NREOLP2 (**b**), NREOLP3 (**c**), REOLP1 (**d**), REOLP2 (**e**), and REOLP3 (**f**). **g**,**h** Heatmaps displaying marker gene expression in epithelial regions (**g**) and immune regions (**h**) across all samples during the inflammatory development of the oral mucosal epithelium. **i-l**, Violin plot of the expression HMGB1 in pyroptotic epithelial cell regions (**i**), IL1B in TREM1$^+$ Mφ regions (**j**), IL17A (**k**), and CSF2 (**l**) in pathogenic Th17 cell regions.

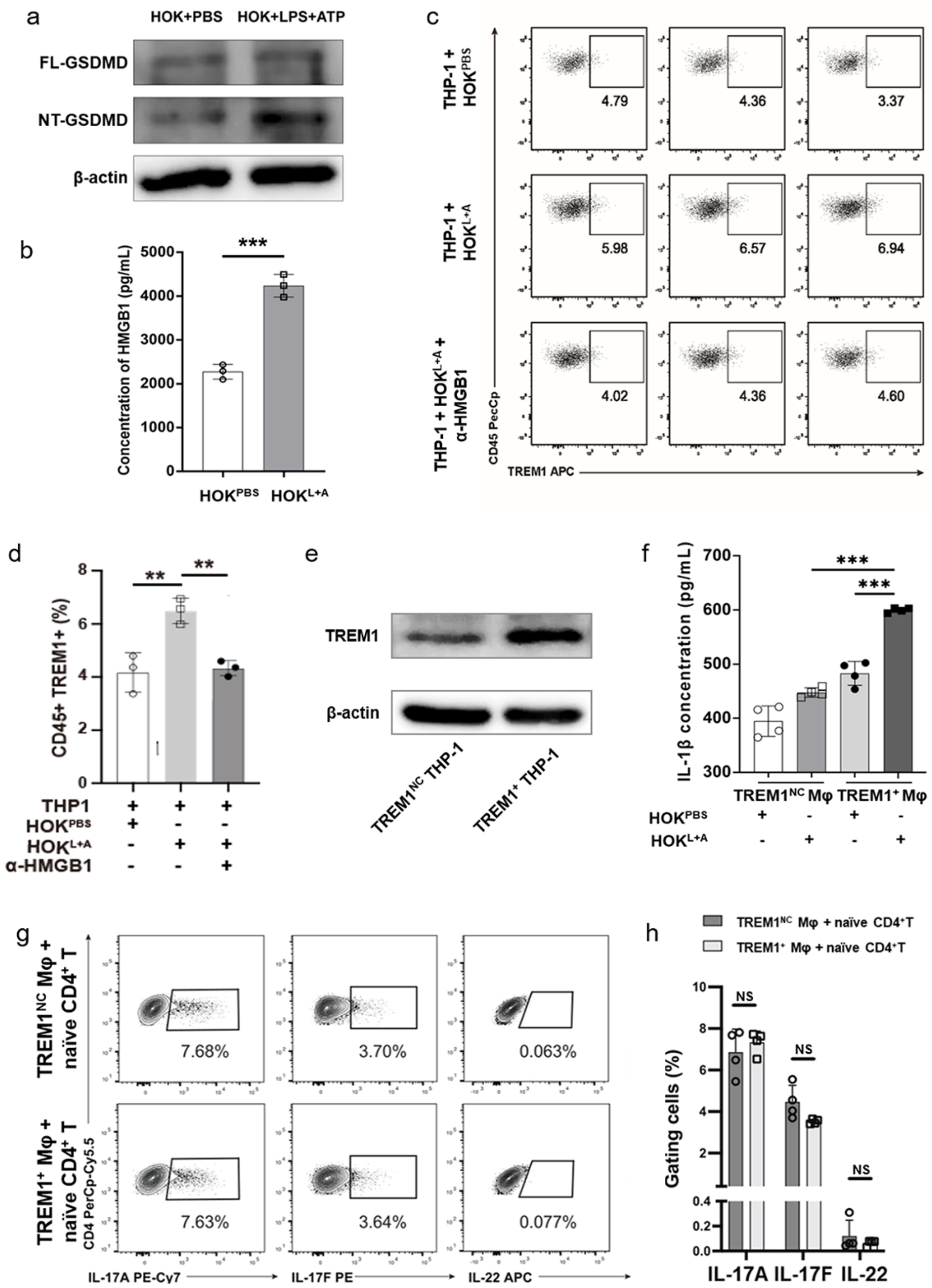

Fig. S3 Cytological experiments elucidated the regulation of TREM1$^+$ Mφ, pathogenic Th17 cells, and pyroptotic epithelial cells. **a**, Western blotting (WB) revealed the protein expression of full-length GSDMD (FL-GSDMD) and N-terminal domain GSDMD (NT-GSDMD) in the pyroptotic epithelial cell model (HOK+LPS+ATP) and normal epithelial cell model (HOK+PBS). **b**, Elisa assay displaying the HMGB1 concentration of the supernatants in pyroptotic epithelial cell model (HOK^{L+A}) and normal epithelial cell model (HOK^{PBS}). **c**,**d** Flow cytometry (**c**) and statistical analysis (**d**) of the cell proportion of TREM1$^+$ CD45$^+$ cell after THP-1 cells co-cultured with the pyroptotic epithelial cell model (HOK^{L+A})/ normal epithelial

cell model (HOK^{PBS}), with/without neutralizing anti-HMGB1 (α-HMGB1). **e**, WB comparing TREM1 protein expression between TREM1-overexpressing THP-1 cells and controls. **f**, Elisa assay describing the concentration of IL-1β in cell co-culture system of pyroptotic epithelial cell model (HOK^{L+A})/ normal epithelial cell model (HOK^{PBS}) with TREM1^{+} Mφ/ TREM1^{NC} Mφ. **g,h**, Flow cytometry (**g**) and statistical analysis (**h**) of the cell proportion of CD4^{+} IL17A^{+} cells, CD4^{+} IL17F^{+} cells, and CD4^{+} IL22^{+} cells after murine naïve CD4^{+} cells co-cultured with the supernatants of TREM1^{+} Mφ, IL6 and TGFβ. Note: **: $p < 0.01$, ***: $p < 0.001$. NS: Not significant.

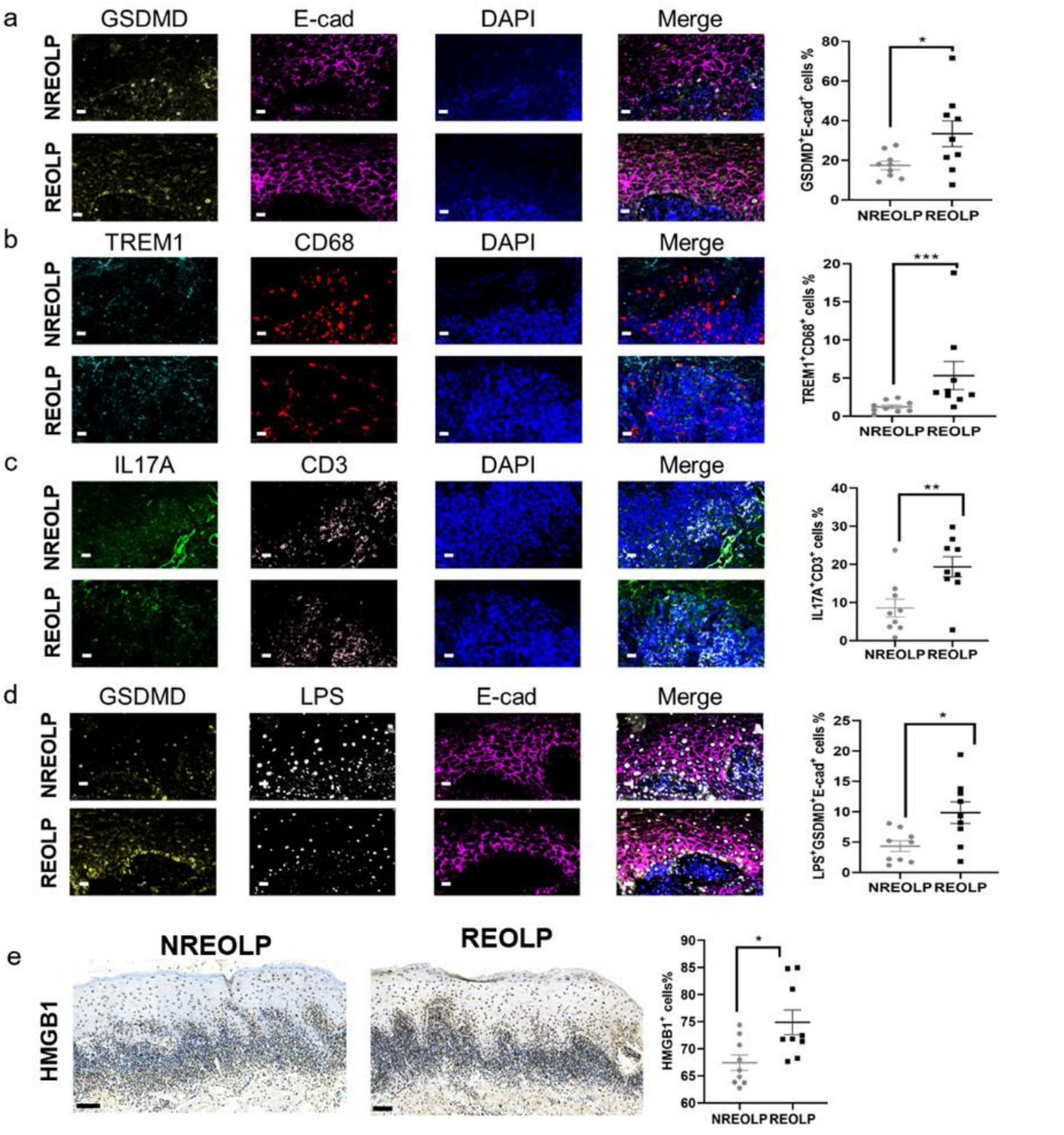

Fig. S4 MIHC revealed the dynamic changes in the spatial distribution of cell types in the inflammatory development of oral mucosal epithelium. a-d, Representative images (left) and statistical analysis (right) of GSDMD^{+} E-cad^{+} cells (**a**), TREM1^{+} CD68^{+} cells (**b**), IL-17A^{+} CD3^{+} cells (**c**), LPS^{+} GSDMD^{+} E-cad^{+} cells (**d**) in the inflammatory development of oral mucosal epithelium. Scale bars: 20 μM. **e**, Representative images of HMGB1 on IHC (**left**), with histologic analysis (**right**). Scale bars: 100 μM. Note: *: $p < 0.05$, **: $p < 0.01$, ***: $p < 0.001$.

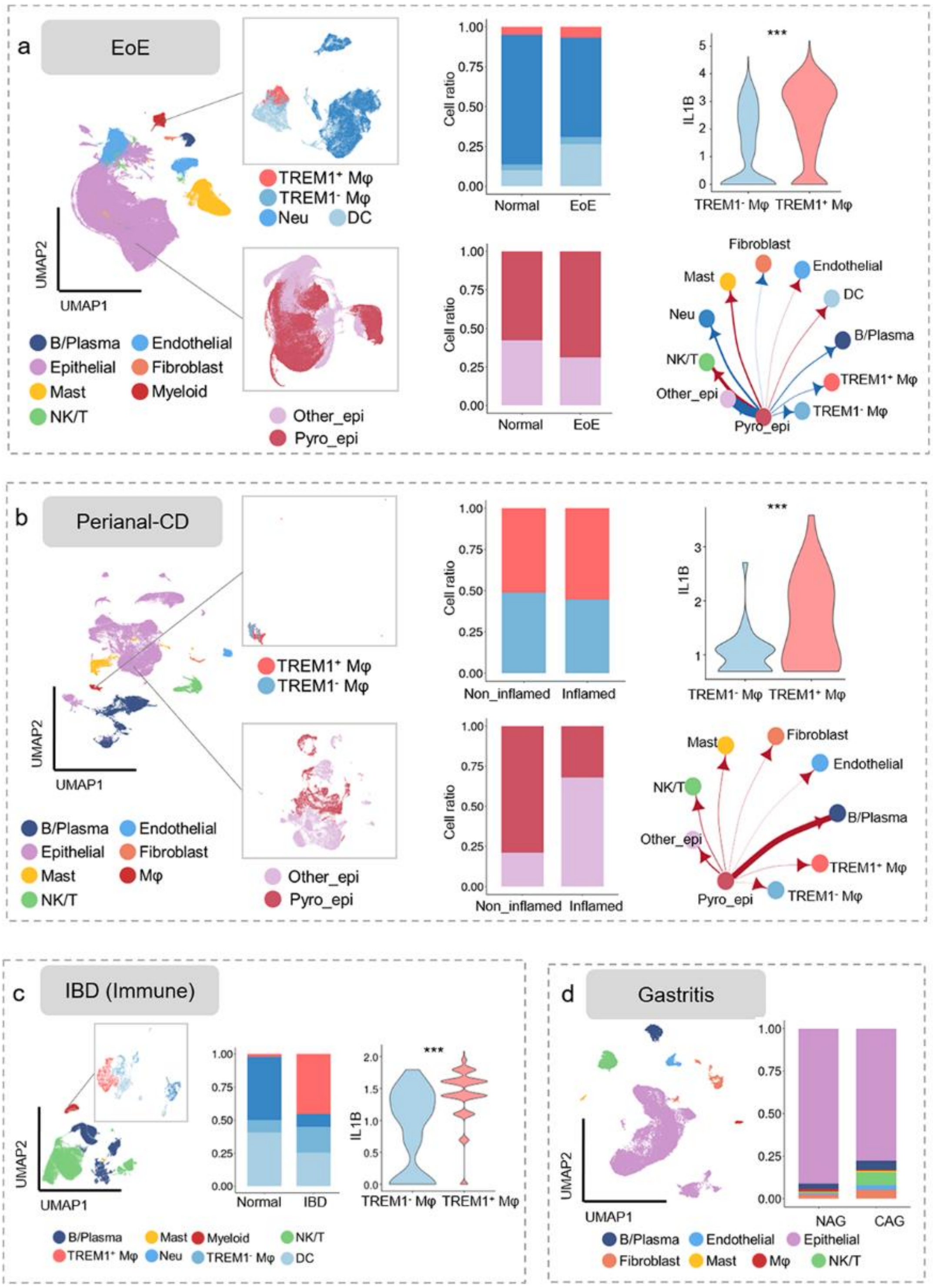

Fig. S5 ORGUAMIA exhibited the significant role of TREM1$^+$ Mφ in the inflammatory development of chronic digestive disorders. a, **b**, The role of epithelial pyroptosis-induced TREM1$^+$ Mφ in EoE datasets (**a**) and perianal-CD dataset (**b**). Left, UMAP plot of cell colors by cell types, myeloid cell/Mφ types, and epithelial cell types. Middle, Histogram showing the cell proportions of myeloid cell/Mφ types (top) and epithelial cell types with disease subtypes (bottom). Top right, violin plot showing the IL1B expression in Mφ subtype. Bottom right, circle plot of the intercellular communication from pyroptotic epithelial cells to other cells. Line width, the intercellular communication strength. Red arrow, the intercellular communication

strength in EoE/inflamed perianal-CD was higher than in normal/non-inflamed perianal-CD. Blue arrow, the intercellular communication strength in normal/non-inflamed perianal-CD was higher than in EoE/inflamed perianal-CD. **c**, Left, UMAP plot of cell colors by cell types and myeloid cell types in IBD datasets only include immune cells. Middle, histogram showing the cell proportions of myeloid cell types with disease subtypes in IBD datasets only include immune cells. Right, violin plot showing the IL1B expression of Mφ subtypes in IBD datasets only included immune cells. **d**, UMAP plot (left) and histogram (right) of cell colors by cell types in gastritis dataset. NAG: non-atrophic gastritis. CAG: chronic atrophic gastritis.

Table S1 Basic information of the clinical follow-up cohort.

Factors		Overall	NREOLP	REOLP	*p*
n		116	94	22	
Age (mean (SD))		41.4 (11.4)	40.7 (11.6)	44.4 (10.3)	0.169
gender (%)	female	75 (64.7%)	63 (67.0%)	12 (54.5%)	0.393
	male	41 (35.3%)	31 (33.0%)	10 (45.5%)	
Smoking (%)	Yes	95 (81.9%)	76 (80.9%)	19 (86.4%)	0.767
	No	21 (18.1%)	18 (19.1%)	3 (13.6%)	
Alcohol (%)	Yes	73 (62.9%)	62 (66.0%)	11 (50.0%)	0.250
	No	43 (37.1%)	32 (34.0%)	11 (50.0%)	

Table S2 Multivariable logistic regression analysis of the clinical follow-up cohort.

Factors		OR (95% CI)	*p*	VIF
Age		1.02 (0.96 - 1.07)	0.593	1.17
Gender	Female	-	-	-
	Male	1.75 (0.43 - 7.25)	0.432	-
Smoking	No	-	-	-
	Yes	0.55 (0.08 - 3.05)	0.514	-
Alcohol	No	-	-	-
	Yes	1.47 (0.41 - 5.11)	0.541	-
Genes		-	-	-
	GSDMD	1.90 (0.27 - 13.04)	0.510	2.97
	HMGB1	2.19 (0.22 - 28.05)	0.515	1.85
	CD68	0.72 (0.10 - 5.16)	0.737	2.22
	TREM1	0.67 (0.26 - 1.59)	0.378	2.39
	IL1B	2.38 (1.19 - 5.17)	0.020	2.19
	IL17A	1.45 (0.60 - 3.37)	0.393	1.70
	CSF2	1.25 (0.53 - 3.04)	0.605	2.45

OR: odds ratio, VIF: variance inflation factor.

Table S3 Collinearity diagnostics based on condition indices and variance decomposition of marker genes.

Condition index	Intercept	CD68	GSDMD	IL1B	TREM1	CSF2	IL17A	HMGB1
1.000	0.000	0.000	0.000	0.000	0.003	0.002	0.004	0.000
3.661	0.000	0.000	0.000	0.001	0.050	0.003	0.342	0.000
5.792	0.000	0.000	0.000	0.001	0.474	0.005	0.479	0.000
8.867	0.000	0.000	0.000	0.001	0.164	0.764	0.094	0.001
18.544	0.003	0.001	0.003	0.982	0.245	0.034	0.037	0.006
44.914	0.163	0.032	0.226	0.000	0.000	0.074	0.024	0.237
54.649	0.004	0.012	0.557	0.005	0.021	0.113	0.005	0.745
105.647	0.830	0.954	0.213	0.010	0.044	0.005	0.014	0.010

Table S4 Ridge regression coefficients and bootstrap - validated statistical metrics for marker genes in the clinical follow-up cohort.

Genes	Ridge regression model	Bootstrap			
	Coefficient	95%CI	Median	Mean	SD
CD68	0.036	0.005-0.064	0.036	0.036	0.015
CSF2	0.043	0.008-0.071	0.042	0.041	0.016
GSDMD	0.045	0.021-0.069	0.044	0.044	0.012
HMGB1	0.042	0.011-0.072	0.042	0.042	0.016
IL17A	0.032	0.003-0.063	0.032	0.032	0.015
IL1B	0.066	0.035-0.090	0.066	0.066	0.014
TREM1	0.039	0.006-0.070	0.039	0.038	0.017

Table S5 Public datasets of ORGUAMIA.

GEO ID	Dataset ID	Patients/Samples	Organ	Diseases	Technique	Source	Note
GSE152042	GSE152042	4/4	Mouth	Periodontitis	scRNA-seq	Tissue	
GSE164241	GSE164241	30/30	Mouth	Periodontitis	scRNA-seq	Tissue	
GSE171213	GSE171213	12/12	Mouth	Periodontitis	scRNA-seq	Tissue	
GSE174609	GSE174609	12/12	Mouth	Periodontitis	scRNA-seq	Blood	
GSE206621	GSE206621	8/8	Mouth	Periodontitis	ST	Tissue	
GSE207502	GSE207502	3/3	Mouth	Periodontitis	scRNA-seq	Tissue	
GSE244515	GSE244515	27/27	Mouth	Periodontitis	scRNA-seq	Blood	
GSE211630	GSE211630	6/6	Mouth	OLP	scRNA-seq	Tissue	
GSE213345	GSE213345	3/3	Mouth	OLP	ST	Tissue	
GSE116130	GSE116130-B	2/3	Mouth	Inflammatory oral mucosa	scRNA-seq	Blood	
GSE116130	GSE116130-T	4/4	Mouth	Inflammatory oral mucosa	scRNA-seq	Tissue	
GSE126250	GSE126250	22/1089	Esophagus	EoE	scRNA-seq	Tissue	Only T cells
GSE201153	GSE201153	10/10	Esophagus	EoE	scRNA-seq	Tissue	
GSE218607	GSE218607	12/31	Esophagus	EoE	scRNA-seq	Tissue	
GSE134520	GSE134520	13/13	Stomach	Gastritis	scRNA-seq	Tissue	
GSE116222	GSE116222	3/9	Gut	IBD	scRNA-seq	Tissue	
GSE125527	GSE125527	15/15	Gut	IBD	scRNA-seq	Tissue	
GSE140123	GSE140123	3/3	Gut	IBD	scRNA-seq	Tissue	
GSE148837	GSE148837	6/6	Gut	IBD	scRNA-seq	Tissue	CD8 T cells
GSE150115	GSE150115	5/5	Gut	IBD	scRNA-seq	Tissue	
GSE153866	GSE153866	2/2	Gut	IBD	scRNA-seq	Tissue	

GSE162335	GSE162335	26/26	Gut	IBD	scRNA-seq	Tissue	
GSE169136	GSE169136	6/11	Gut	IBD	scRNA-seq	Tissue	Innate lymphoid cells, NK cells and T cells
GSE182270	GSE182270	9/9	Gut	IBD	scRNA-seq	Tissue	
GSE202052	GSE202052	27/27	Gut	IBD	scRNA-seq	Tissue	
GSE209832	GSE209832	11/11	Gut	IBD	scRNA-seq	Tissue	
GSE214695	GSE214695	18/18	Gut	IBD	scRNA-seq	Tissue	
GSE221987	GSE221987	4/4	Gut	IBD	scRNA-seq	Tissue	
GSE231993	GSE231993	8/12	Gut	IBD	scRNA-seq	Tissue	
GSE234713	GSE234713	9/9	Gut	IBD	ST	Tissue	
GSE242086	GSE242086	6/12	Gut	IBD	scRNA-seq	Tissue	
GSE252122	GSE252122	2/2	Gut	IBD	scRNA-seq	Blood	
GSE225199	GSE225199	13/13	Gut	IBD	scRNA-seq	Tissue	

EoE: eosinophilic esophagitis, IBD: inflammatory bowel disease, scRNA-seq: single-cell RNA sequencing, ST: spatial transcriptome.

File S1 The authentication report of the HOK cell line.

Analysis of the results

一、Test result：

1. The results of the negative and positive control match expectations.
2. The STR profiles of the cell line sample are in the attached table and figure.

Genetic Site	Cellosaurus				Customer sample			
(Locus)	Database profile：				Query profile:HOK			
Amelogenin					X			
D5S818					12	13		
D13S317					12			
D7S820					10	12		
D16S539					12			
vWA					17	18	19	
TH01					7	9		
TPOX					8	10		
CSF1PO					12	13		
The number of matched peaks								
The number of peaks in the database								
Percent match between the query and the database profile：								

二、Analysis description：

The DNA of the cell was amplified and the map was clear and the typing result was good.

三、Test results：

HOK：①The results of STR typing of the cell DNA of this strain showed that the vWA has three alleles, and the other loci have no multiple alleles.No cross-contamination of other human cell line is found. ②No cells with a matching degree of more than 80% with their STR typing data were found in the Cellosaurus cell bank. The specific comparison results are shown in the attached drawings.

（This result is only responsible for this inspection material）

Technician: Xiaping Fan
Check: Lili Zhu
Person in Charge: Shanlin Sun

Issue date:: 2023/03/01

Inspection material

Table 1. Sample information

sample marker	Test number	sample type
HOK	CJ23-CDKQ-HC-02	Cell Freezing Medium

Date of appointment: 2023.02.22

Date of sample receipt: 2023.02.22

Test date: 2023.02.24

Delegate requirements：STR typing test to check the cell against the Cellosaurous database and monitor whether there is cross-contamination of different kinds of cells.

Detection method:

1. PCR is amplified with 21 STR Multi-amplification Kit;
2. PCR products are assayed with ABI 3130xl DNA Analyzer(Applied Biosystems®).
3. Data were analyzed using GeneMapperIDX software and then compared with the ATCC 、DSMZ、JCRB and COG databases for reference matching.

Testing base:

Refer to the cell identification standard (ASN-0002-2011) issued by the International Committee for Cell Identification (ICLAC): STR detection method is used for the identification of human cells. This method suggests that at least 8 STR gene loci and one sex locus should be included for STR identification of human cells.

Notes：

1. According to the cell STR identification criteria established by the international committee for cell identification (ICLAC), cell lines with ≥80% match are considered to be related or derived from a common ancestry. Cell lines with between a 55% to 80% match require further profiling for authentication of relatedness.

2. The effective peak was the real PCR band; Small peaks and nonspecific bands were ignored in the calculation.

3. This matching degree is calculated according to the calculation rules of ATCC by default, and DSMZ's calculation rules in a small number of cases. The commission can only propose in advance if there is personalized demand.

4. If the client's cell samples are stem cells, immune cells, primary cells and other cells and have not been submitted to the public database, the comparison of STR data with the database is meaningless.

5. STR data is compared with Cellosaurus database by default, which is the most authoritative and comprehensive comparison source of cell line STR database at present, and it collects human and mouse cell STR data published in all databases or literatures such as ATCC, DSMZ, JCRB, ECACC, COG, CBA, KCLB. If there are other requirements for personality comparison data, the source of STR data comparison of the tested sample should be indicated in advance.

6. There is only one number in locus typing, indicating that the locus is homozygous. The locus has two different Numbers, indicating that it is a heterozygote.

Table: STR profiles of “HOK”cell line

HOK				
Marker	Allele 1	Allele 2	Allele 3	Allele 4
D19S433	13	15		
D5S818	12	13		
D21S11	30			
D18S51	16	17		
D6S1043	13	21.3		
AMEL	X			
D3S1358	17			
D13S317	12			
D7S820	10	12		
D16S539	12			
CSF1PO	12	13		
Penta D	9	11		
D2S441	10	12		
vWA	17	18	19	
D8S1179	9	14		
TPOX	8	10		
Penta E	13	14		
TH01	7	9		
D12S391	20			
D2S1338	16	23		
FGA	23	24		

File S2 The authentication report of the THP-1 cell line.

Analysis of the results

一、Test result：

1. The results of the negative and positive control match expectations.
2. The STR profiles of the cell line sample are in the attached table and figure.

Genetic Site	Cellosaurus				Customer sample			
(Locus)	Database profile：THP-1				Query profile:THP-1			
Amelogenin	X	Y			X	Y		
D5S818	11	12			11			
D13S317	13				13			
D7S820	10				10			
D16S539	11	12			11	12		
vWA	16				16			
TH01	8	9.3			8	9.3		
TPOX	8	11			8	11		
CSF1PO	11	13			13			
The number of matched peaks								11
The number of peaks in the database								24
Percent match between the query and the database profile：								91.67%

二、Analysis description：

The DNA of the cell was amplified and the map was clear and the typing result was good.

三、Test results：

THP-1：①The results of STR typing of the cell DNA of this strain showed that there is no multiple allele phenomenon in each locus.No cross-contamination of other human cell line is found. ②The submitted profile is a 91.67% match for the following human cell line in the Cellosaurus STR database (8 core loci plus Amelogenin):THP-1。

（This result is only responsible for this inspection material）

Technician: Xiaping Fan
Check: Lili Zhu
Person in Charge: Shanlin Sun

Issue date:: 2023/03/15

Inspection material

Table 1. Sample information

sample marker	Test number	sample type
THP-1	CJ23-CDKQ-HC-03	Cell Freezing Medium

Date of appointment: 2023.03.08

Date of sample receipt: 2023.03.08

Test date: 2023.03.10

Delegate requirements： STR typing test to check the cell against the Cellosaurous database and monitor whether there is cross-contamination of different kinds of cells.

Detection method:

1. PCR is amplified with 21 STR Multi-amplification Kit;
2. PCR products are assayed with ABI 3130xl DNA Analyzer(Applied Biosystems®).
3. Data were analyzed using GeneMapperIDX software and then compared with the ATCC 、DSMZ、JCRB and COG databases for reference matching.

Testing base:

Refer to the cell identification standard (ASN-0002-2011) issued by the International Committee for Cell Identification (ICLAC): STR detection method is used for the identification of human cells. This method suggests that at least 8 STR gene loci and one sex locus should be included for STR identification of human cells.

107

Notes：

1. According to the cell STR identification criteria established by the international committee for cell identification (ICLAC), cell lines with ≥80% match are considered to be related or derived from a common ancestry. Cell lines with between a 55% to 80% match require further profiling for authentication of relatedness.
2. The effective peak was the real PCR band; Small peaks and nonspecific bands were ignored in the calculation.
3. This matching degree is calculated according to the calculation rules of ATCC by default, and DSMZ's calculation rules in a small number of cases. The commission can only propose in advance if there is personalized demand.
4. If the client's cell samples are stem cells, immune cells, primary cells and other cells and have not been submitted to the public database, the comparison of STR data with the database is meaningless.
5. STR data is compared with Cellosaurus database by default, which is the most authoritative and comprehensive comparison source of cell line STR database at present, and it collects human and mouse cell STR data published in all databases or literatures such as ATCC, DSMZ, JCRB, ECACC, COG, CBA, KCLB. If there are other requirements for personality comparison data, the source of STR data comparison of the tested sample should be indicated in advance.
6. There is only one number in locus typing, indicating that the locus is homozygous. The locus has two different Numbers, indicating that it is a heterozygote.

Table: STR profiles of "THP-1" cell line

THP-1				
Marker	Allele 1	Allele 2	Allele 3	Allele 4
D19S433	12.2	13		
D5S818	11			
D21S11	30	31.2		
D18S51	13	14		
D6S1043	14			
AMEL	X	Y		
D3S1358	15	17		
D13S317	13			
D7S820	10			
D16S539	11	12		
CSF1PO	13			
Penta D	10	12		
D2S441	10	11		
vWA	16			
D8S1179	10	14		
TPOX	8	11		
Penta E	11	15		
TH01	8	9.3		
D12S391	19			
D2S1338	17	18		
FGA	24	25		
