# Supplementary figures and images for: Epithelial pyroptosis-induced TREM1+ macrophages activate Th17 cells to accelerate oral mucosal inflammation
Source: Cell Death Discov. 2025 Nov 29;12:26. doi: 10.1038/s41420-025-02853-7 (PMC12811386; doi:10.1038/s41420-025-02853-7)

# Full Length-GSDMD

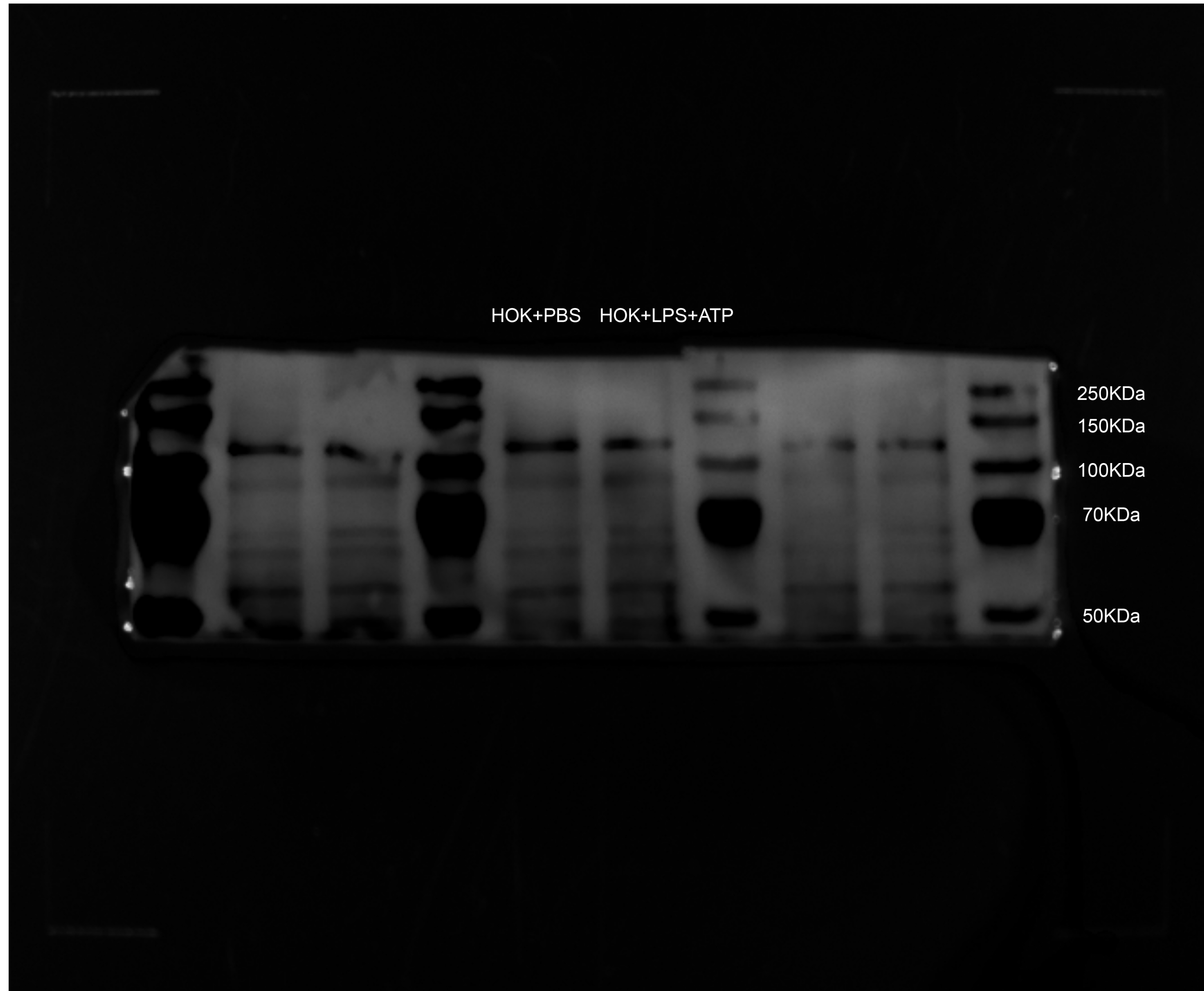

$\beta$ -actin

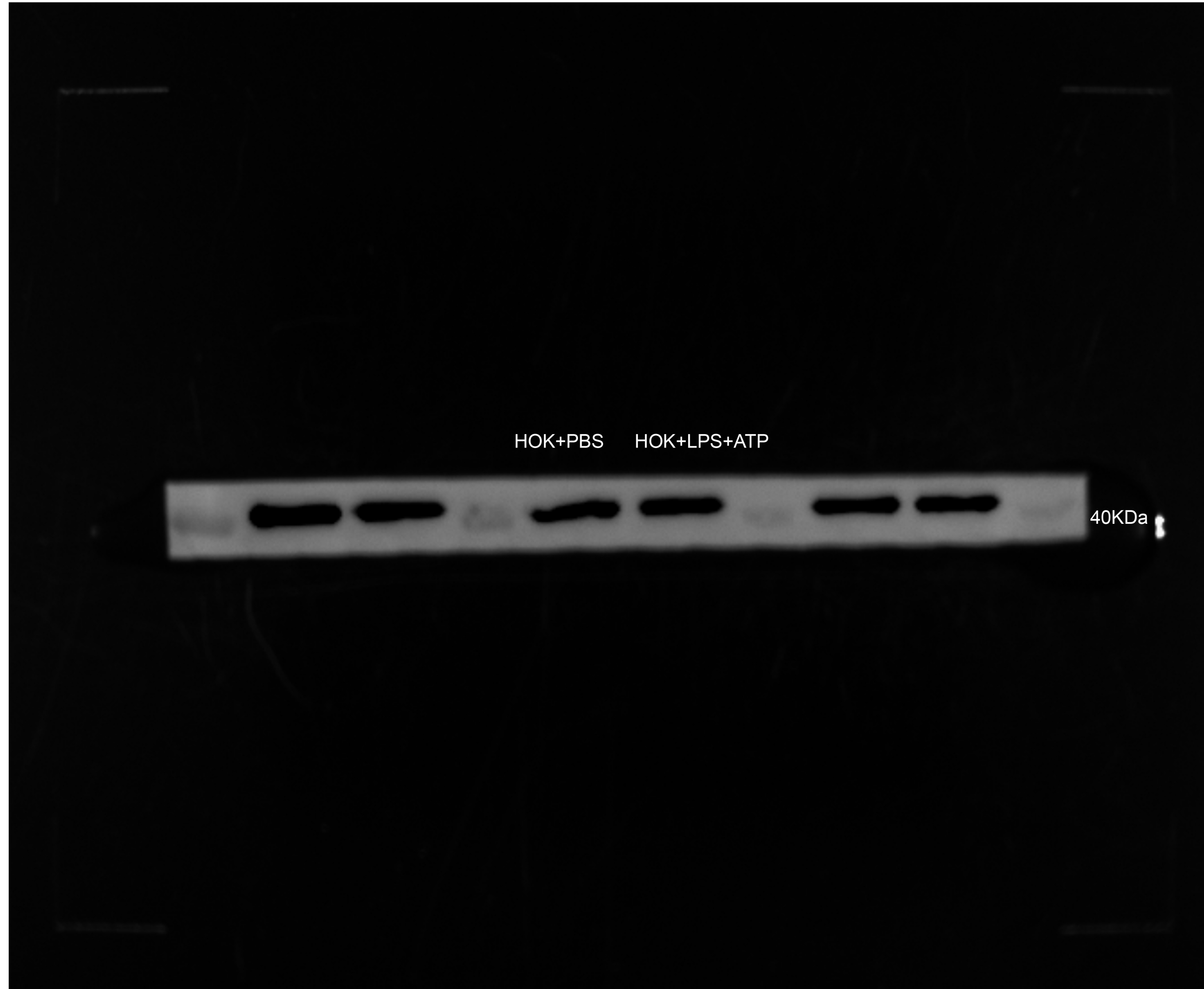

# NT-GSDMD

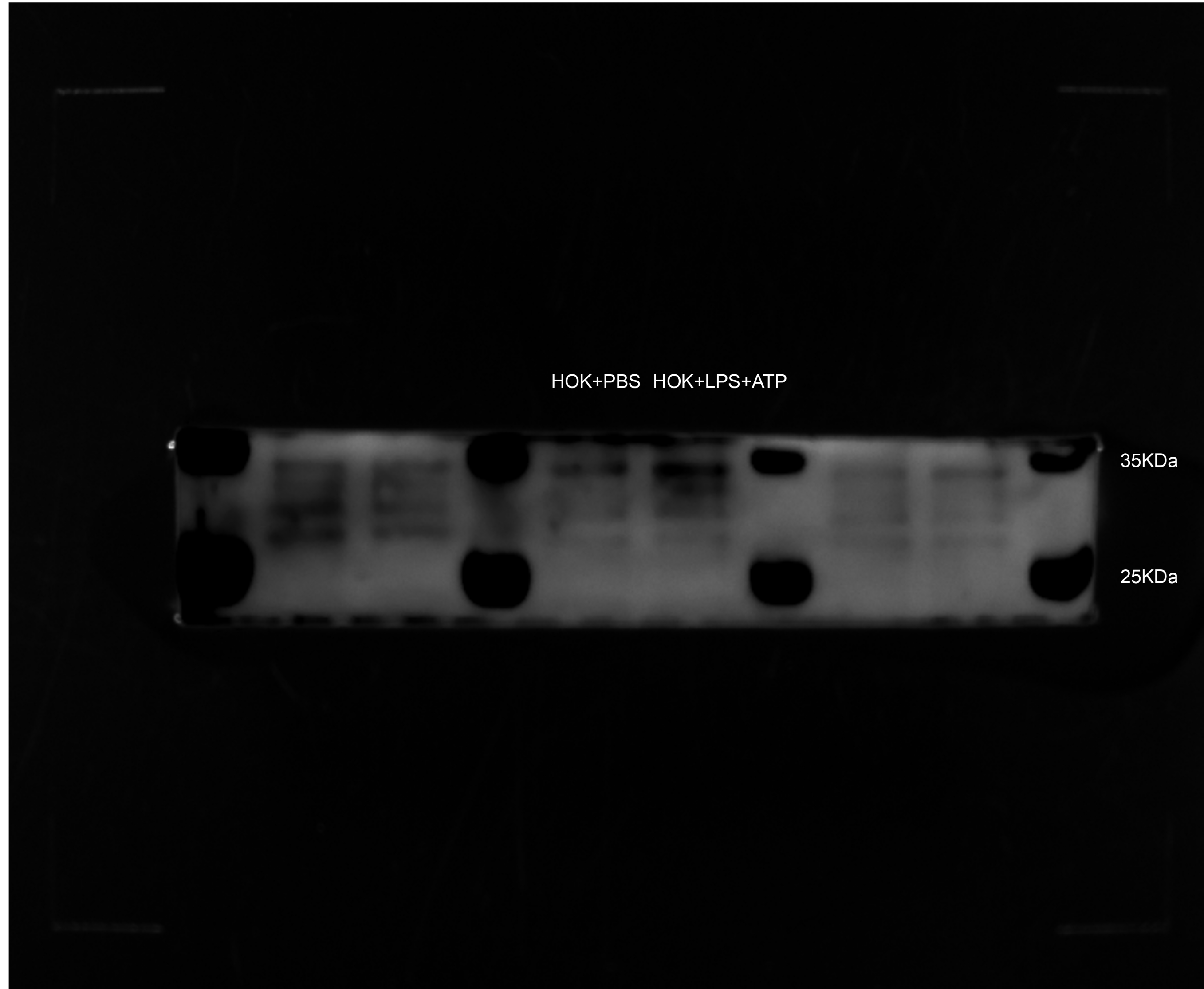

# TREM1

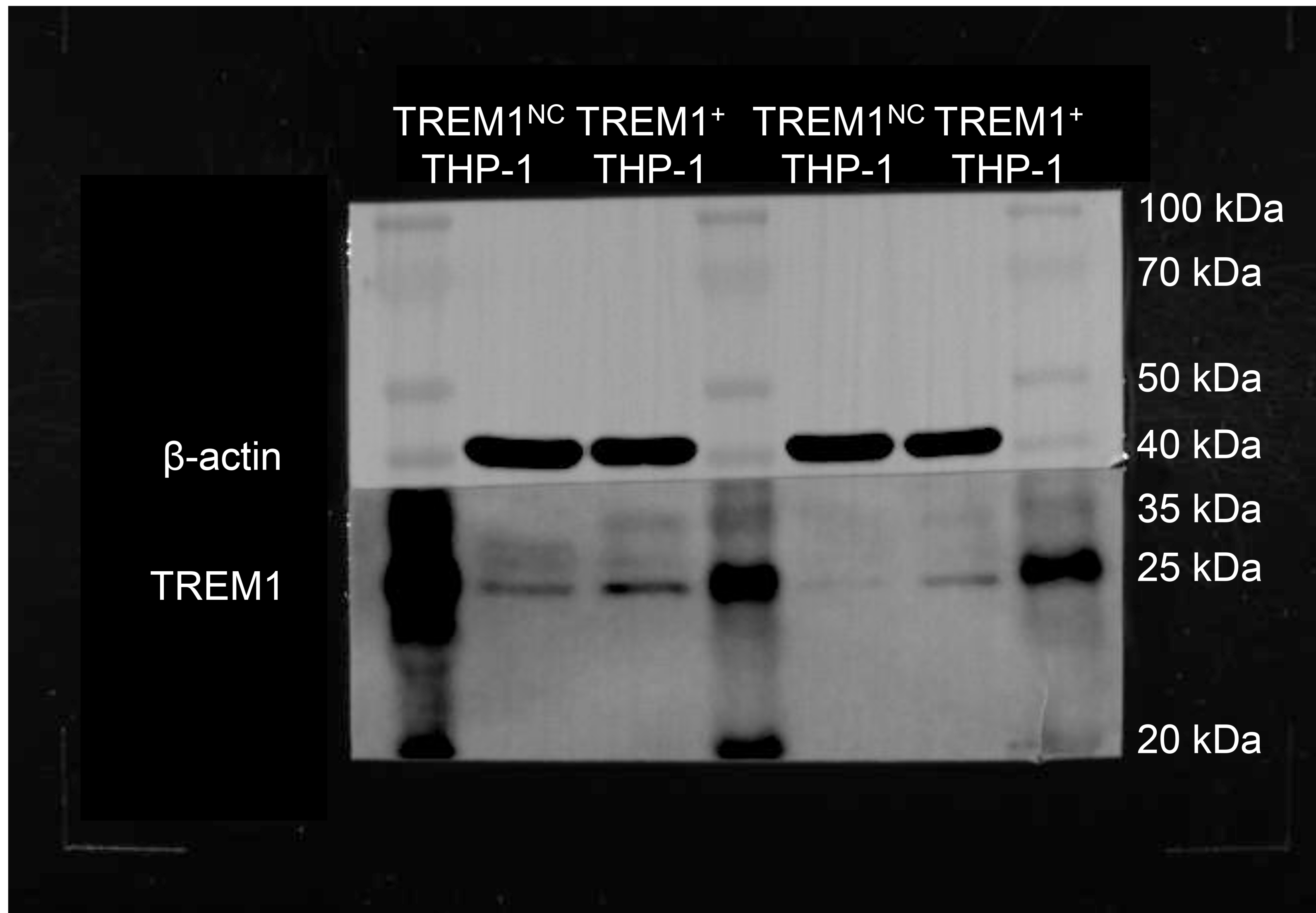

Supplement: Supplementary file 2 — WB [file 41420_2025_2853_MOESM2_ESM.pdf]
